# Gene expression profiling and pathway analysis in acute myeloid leukaemia-normal karyotype patients

**DOI:** 10.1371/journal.pone.0328911

**Published:** 2025-09-05

**Authors:** Angeli Ambayya, Rozaimi Razali, Sarina Sulong, Yee Yee Yap, Veena Selvaratnam, Jameela Sathar, Rosline Hassan

**Affiliations:** 1 Department of Haematology, School of Medical Sciences, Health Campus, Universiti Sains Malaysia, Kubang Kerian, Kelantan, Malaysia; 2 Clinical Haematology Referral Laboratory, Haematology Department, Hospital Ampang, Ampang, Selangor, Malaysia; 3 Department of Biomedical Sciences, College of Health Sciences, QU Health, Qatar University, Doha, Qatar; 4 Human Genome Centre, School of Medical Sciences, School of Medical Sciences, Health Campus, Universiti Sains Malaysia, Kubang Kerian, Kelantan, Malaysia; 5 Hospital Universiti Sains Malaysia, Health Campus, Universiti Sains Malaysia, Kelantan, Malaysia; European Institute of Oncology, ITALY

## Abstract

Acute myeloid leukaemia-normal karyotype (AML-NK) exhibits heterogeneity in expression profiles, influencing the treatment response and survival outcome. Transcriptome sequencing allows a comprehensive analysis of differentially expressed genes (DEGs) and dysregulated pathways in AML-NK, shedding light on the molecular mechanisms and their implications in patients’ management. DEG analyses utilising transcriptome sequencing were conducted using a customised DESeq2 pipeline on 51 AML-NK patients at diagnosis (DX), 12 AML-NK patients who attained first remission (CR1) and 12 healthy controls. The transcriptomic sequencing of AML-NK compared to healthy controls revealed 5,126 DEGs, comprising 85.8% coding genes and 14.2% non-coding elements across 37 pathway categories. The AML-NK DX versus CR1 identified 5,621 DEGs consisting of 84.7% coding genes and 15.3% non-coding elements affecting 20 categories of pathways. Gene set enrichment analysis in this study revealed consistent upregulation of proliferative pathways, including cell cycle and DNA replication. In contrast, immune-related pathways, such as cytokine-cytokine receptor interactions and MHC antigen presentation pathways, were downregulated. Overexpression of oncogenes (*FLT3, MYB, DNMT3B, and MYCN*) in DX vs CR1 samples reinforces their usefulness in minimal residual disease monitoring, especially in AML-NK with no genetic aberrations. These findings reiterate the known hallmarks of cancers and validate the transcriptomic dysregulation in the pathogenesis of AML-NK. The robustness of the transcriptome sequencing findings was confirmed by RT-qPCR validation of six genes that were not reported in AML-NK patients. The comprehensive analyses of pathways with dysregulation of a myriad of genes led to an understanding of AML-NK pathogenesis and highlighted the markers for minimal residual disease. In summary, this study performed the first transcriptome-wide analysis of AML-NK in a Malaysian cohort and underscored pathways that are candidates for therapeutic interventions.

## Introduction

Acute myeloid leukaemia (AML) is a heterogeneous haematological malignancy characterised by the clonal expansion of immature myeloid cells, generally due to genomic aberrations that regulate haematopoiesis [1]. Based on conventional cytogenetic analysis, AML-normal karyotype (AML-NK) is the absence of any detectable chromosomal abnormalities. The diagnosis and prognostication of AML-NK remain challenging due to the lack of cytogenetic markers for risk stratification [2–4]. Despite the absence of detectable chromosomal abnormalities, cryptic mutations and gene expression profiles that can influence disease prognosis, therapy response, and survival outcomes are hallmarks of AML-NK [5,6]. Transcriptome sequencing has emerged as a powerful tool to characterise the transcriptomic landscape of AML, particularly in cases with normal karyotype, to identify differential gene expression that may underlie disease development and progression [7,8].

Whole transcriptome sequencing for DEG profiling has been revolutionised for over a decade. In AML, gene expression studies are useful in identifying the subtypes and patient stratification and providing new insights into the mechanism of leukaemogenesis and the development of therapeutic targets. Researchers have identified unique gene expression signatures associated with chromosomal aberrations, normal karyotypes, and mutational status of genes such as *NPM1* using supervised and unsupervised learning algorithms [9,10]. Distinct gene expression signatures enabled the identification of prognostically important subtypes of AML, particularly in AML-NK. Besides that, DEG studies in older AML patients have revealed unique signatures which not only impacted the prognosis but also showed association with genes involved in proliferation, apoptosis, dysregulation of oncogenic signalling pathways, altered tumour environment and signatures of chemotherapy sensitivity [9,10].

In AML-NK, transcriptome sequencing has revealed a distinct transcriptomic profile that can be linked to different biological processes and clinical outcomes. Key findings from studies focusing on differential gene expression in AML-NK include gene expression alteration in signalling pathways, epigenetic regulators, and immune-related genes [11–13]. DEG profiling through transcriptome sequencing in AML-NK has several clinical implications in terms of patient stratification into different risk groups, identification of altered signalling pathways providing a basis for developing targetted therapies and development of immunotherapeutic strategies.

While transcriptome sequencing has revolutionised the understanding of gene regulation in AML-NK, only a few studies have been reported among the Asian populations [14–16]. Some studies reported transcriptome sequencing in the Chinese AML cohort that reported prognosis-related gene signatures, and another study focussed on subtype M2 AML patients [14,15]. Only one local study using transcriptome sequencing on an AML paediatric cohort was reported, including three patients at diagnosis, remission and relapse [16]. This study conducted DEG profiling of AML-NK patients versus healthy control groups and AML-NK patients at initial diagnosis and after the first complete remission. Upon discovering unique DEG profiles for each subgroup, the functional and pathway enrichment profiles were interrogated by GSEA analysis using WebGelstalt (version 2019) [17]. The comprehensive analyses of critical pathways led to understanding the AML-NK pathogenesis. To our knowledge, this is the first and most comprehensive analysis of DEG profiles of AML-NK patients in a Southeast Asian AML-NK cohort.

## Materials and methods

### Study design

This study included a cohort of 51 AML-NK patients diagnosed at Hospital Ampang (HA), Selangor, and Hospital Universiti Sains Malaysia (HUSM), Kubang Kerian, from 2013 to 2017. Sample size calculation is described in S I. AML-NK patients were chosen based on the G-banding karyotype analysis of 20 metaphases of chromosomal spreads acquired from bone marrow aspirates. The subject inclusion and exclusion criteria are listed in S II. DEG profiles of AML-NK patients were compared at diagnosis (DX) and after the first complete remission (CR1) in 12 patients alive during the study period. CR1 was described as patients who have attained remission following the completion of chemotherapy cycles (induction and consolidation) based on morphological leukaemia-free status (bone marrow blast count <5% in an aspirate with spicules and with a count of ≥200 nucleated cells, no blast with Auer rods and no extramedullary disease and leukaemia blast detected in flow cytometry immunophenotyping analysis, absolute neutrophil count (ANC) >1 x 10^9^/L, platelets ≥100 x 10^9^/L [18,19]. The control sample size was determined based on RNA sequencing application and best practice guidelines recommending 6–12 biological controls to identify significantly differentially expressed genes [20]. Hence, in this study, 12 healthy adult controls (aged between 18 and 60 years old) based on gender (six males and six females) and proportion to ethnicity (4 Malays, 4 Chinese, and 4 Indians) were included.

Ethical approvals were obtained from the Medical Research Ethics Committee (MREC) (NMRR ID: 17-1929-36614) and the Human Research Ethics Committee of Universiti Sains Malaysia (JEPeM) (USM/JEPeM/USM/JEPeM/21010107). Patient samples were retrieved from the respective laboratories: HA 1^st^ January 2018− 15^th^ March 2021) and HUSM (15^th^ June 2021− 14^th^ June 2022). All individuals and their legal guardians provided written informed consent wherever necessary in this study, as per approvals obtained.

### Sequencing protocols

The protocol for preparing the library for transcriptome sequencing was conducted using the SureSelect Strand-Specific RNA Library Prep designed for Illumina Multiplexed Sequencing. The sequencing was done on the NovaSeq 6000 platform (Illumina, Inc., San Diego, California). Total RNA was extracted from the patient’s bone marrow aspirates using the QIAamp® RNA Blood Mini kit, and the concentration and purity of the RNA were evaluated with a Nanodrop ND-1000 UV-VIS spectrophotometer. The RNA integrity number (RIN) was determined using the Agilent RNA 6000 Nano Kit on the Agilent 2000 Bioanalyzer, ensuring all samples had a RIN greater than 7. The library preparation process followed the same SureSelect protocol, and the NovaSeq 6000 platform was employed for sequencing. The library concentration was visualised via electropherogram, showing a single peak corresponding to fragment sizes between 200–600 bp. Transcriptome sequencing was conducted at Theragen Etex Inc (Seoul, Korea) with Illumina’s NovaSeq 6000, yielding 150 bp paired-end reads with approximately 200 million reads per sample. The raw data were processed using internal pipelines, starting with quality control of the sequencing results through FastQC (version 0.11.8). Low-quality reads with a Phred score below Q20, along with adapters and poly G sequences, were removed using the FastP tool. The cleaned reads were then aligned to the human reference genome (version 38, Ensembl) using the spliced transcripts alignment to a Reference (STAR) software (version 2.7.1a).

### DEG analysis using DESeq2

DEG pipeline in this study utilised featureCounts (version 2.0.0) and DESeq2 (version 1.26.0) [21]. FeatureCounts performed read summarisation from the transcriptome sequencing reads. Prealignment data processing and alignment to the reference genome (GRCh38.p13) were conducted. BAM files derived post-alignment using the STAR aligner were used as input for the DEG pipeline. The DEG pipeline is attached in S III. DEGs that passed the following filters were classified as significant: p-adjusted value (padj) <0.01, log fold change (lfc) >1 and <−1 and basemean >100. These filters were based on previous validation studies [22,23]. The DESeq2 integrated tools generated MA plots, volcano plots, correlations, and hierarchical heatmaps. This study conducted two DEG analyses: AML-NK at DX (n = 51) vs healthy controls (n = 12) and AML-NK at DX (n = 12) vs CR1 (n = 12). A cut-off of approximately 10% of the total DEGs discovered in the AML-NK Dx vs healthy controls was studied by a comprehensive literature search using several databases that include the National Center for Biotechnology Information (NCBI) database, The Human Gene Database (GeneCards), LNCipedia (for non-coding RNAs) and Google Scholar was performed (last access date: 19^th^ April 2025). DEGs that were not published in these databases were considered novel.

### Functional enrichment analysis by pathway analysis

Websgelstalt (version 2019) was utilised to perform gene set enrichment analysis (GSEA) for pathway analysis (PA) [24]. The mean and median normalised data between AML-NK (DX) and healthy controls and AML-NK (DX vs CR1) were tested with an independent T-test to identify the most significant DEG between these two groups. The most significant short-listed DEGs were selected for downstream functional enrichment analyses using the GSEA function on Websgelstalt. Functional enrichment analysis using GSEA for pathways was carried out using Websgelstalt with customised filtering as follows: (i) minimum of 5 genes for a category, (ii) Benjamini–Hochberg multiple test adjustment, (iii) each category was weighted based on the p-value, false discovery rate (FDR), and the most significant categories were included. The Kyoto Encyclopaedia of Genes and Genomes (KEGG) database was used to identify significantly affected pathways for the DEGs in each group. The cut-offs used for PA were normalised enrichment score (NES) ≥1, *p*-value <0.05 and false discovery rate of ≤0.05.Validation using qPCR for novel DEGs in AML-NK versus healthy group

Reverse transcription-quantitative real-time PCR (RT-qPCR) was performed on the six most significant DEGs not reported in AML (*TDP2, STK40, PACS1, OAZ2, TCF20* and *CRISPLD1),* which were selected from the DEG profile of AML-NK versus healthy controls based on the following filters: p-adjusted value (padj) <0.01, log fold change (lfc) >1 and <−1 and basemean >100. The RT-qPCR protocols are described in detail in S IV.

### Statistical analysis

Normality tests for continuous variables (such as age, haemoglobin, WBC, platelet count, and overall survival) were conducted using the Shapiro-Wilk test when the sample size was below 50 and the Kolmogorov-Smirnov test when the sample size exceeded 50. A p-value of less than 0.05 indicates a non-normal distribution of the data. The results were presented as mean ± standard deviation (SD) for normally distributed data, while median and interquartile range (IQR) were used for non-normally distributed data. A T-test was used to compare two groups with normally distributed data, while an analysis of variance (ANOVA) was used to compare more than two groups. Nonparametric analyses employed the independent-samples Kruskal-Wallis test for multiple group comparisons, followed by post hoc analysis and the Mann-Whitney U test to compare two groups. Post hoc testing following a Kruskal-Wallis test utilised Dunn-Bonferroni nonparametric comparisons. A significance threshold of 0.05 was established to determine statistical significance. The statistical analyses were done using the Statistical Package for the Social Sciences (SPSS) version 26 (IBM, USA).

## Results

### Patient characteristics

A total of 51 AML-NK patients participated in this study, which included 27 males (53%) and 24 females (47%), resulting in a male-to-female ratio of 1.1:1.0. The ages of the AML-NK patients ranged from 15.45 to 70.58 years, with a median age of 47 years (IQR: 22–57) for males and 49 years (IQR: 32–57) for females. Overall, 80% (41 out of 51) of the AML-NK patients were under 60, while approximately 20% (10 out of 51) were over 60. Patient information can be found in S V.

### Transcriptome sequencing quality control data

The transcriptome library preparation and the transcriptome sequencing batch runs are summarised in S VI. The transcriptome sequencing quality control data, including the FASTP (version 0.21.0) quality filtering and alignment with the reference genome using the STAR software (version 2.7.1a), are summarised in S VI [25,26]

### DEG profiles of AML-NK patients versus healthy control groups

Unsupervised clustering of the AML-NK patients (n = 51) against the healthy controls (n = 12) revealed distinct gene expressions in profiles. The principal component analysis (PCA), volcano plot and a hierarchical clustering heatmap are available in S VII. This study discovered 5126 dysregulated genes, encompassing 85.8% (n = 4396/5126) coding genes and 14.2% (n = 730/5126) non-coding elements after filtering based on the following: p-adj < 0.01, log2FoldChange >1 and <−1, and BaseMean >100. About 52.3% (n = 2682/5126) of these genes were downregulated, and 47.7% (n = 2444/5126) were upregulated.

A comprehensive literature search on the top 600 DEGs revealed that 87.8% (n = 527/600) were coding genes, and 12.2% (n = 73/600) were non-coding elements (last update date: 19th April 2025). Of the 527 coding DEGs, 35.9% were reported in AML, whereas 15.7% were novel in AML-NK, and about 48.4% (n = 255/527) coding DEGs were novel for AML but were previously reported in other disorders. The majority of the non-coding elements were lncRNAs (6.8%, n = 41/600), followed by lincRNAs (2.8%, n = 17/600), pseudogenes (1.8%, n = 11/600), ncRNA (0.5%, n = 3/600) and miRNA (0.2%, n = 1/600). These findings are summarised in S VIII.

**Fig 1:**
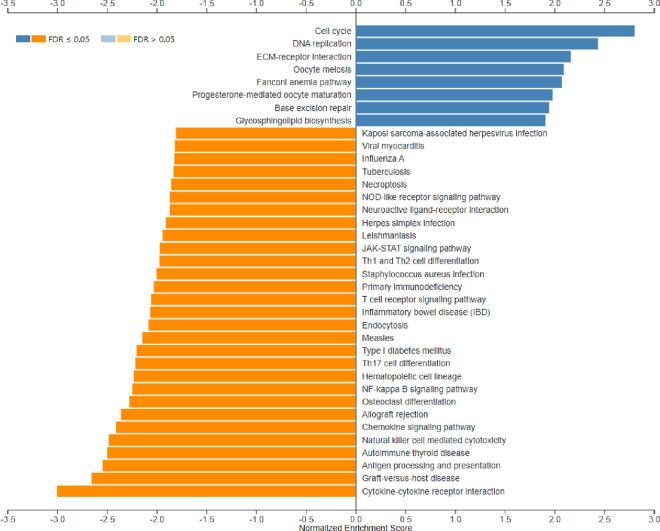
GSEA analysis of AML-NK patients vs healthy controls bar chart. The bar chart depicts significantly enriched pathways in the GSEA analysis of AML-NK patients versus healthy controls. The bar width equals the enrichment ratio, with blue indicating positively related categories and orange indicating negatively related categories in the GSEA analysis. This figure was generated using the WebGestalt tool [24].

Functional enrichment analysis (PA) using the GSEA function on the WebGestalt tool was performed using the 5,126 dysregulated genes. The cut-offs used for the WebGestalt tool are described in the methodology. A total of 37 categories of pathways were affected, as shown in Fig 1.

**Fig 2:**
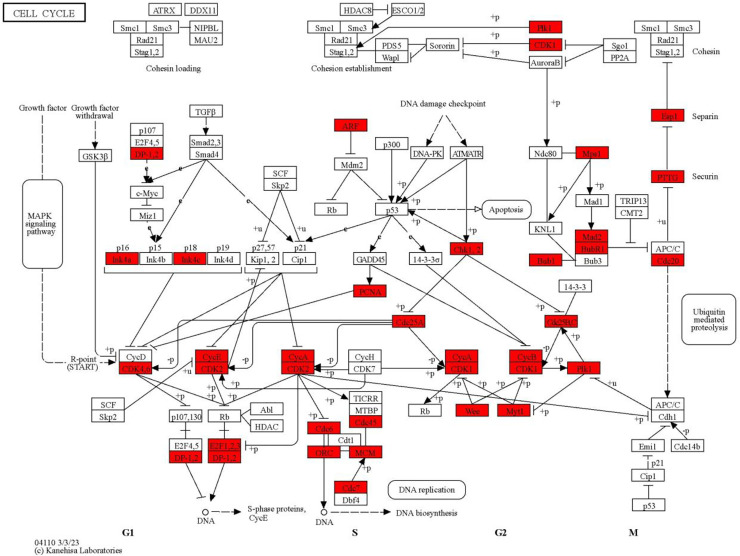
Cell cycle pathway (hsa04110). In this analysis, 56 genes were affected with a NES of 2.81. The image was generated using KEGG Mapper (copyright permission obtained from Kanehisa Laboratories) [27]. All the upregulated genes (red boxes) in the cell cycle pathway are seen in different phases of cell cycles, including G1-phase, S-phase, G2-phase, and M-phase.

**Fig 3:**
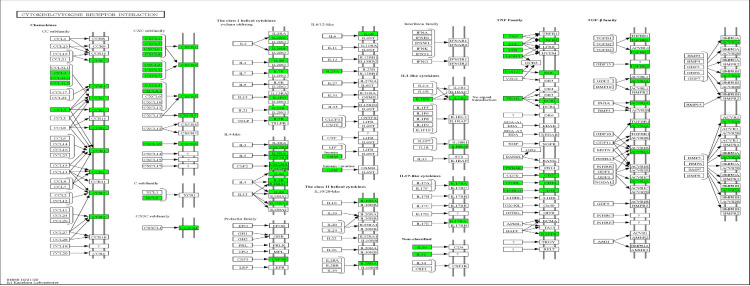
Cytokine-cytokine receptor interaction (hsa04060). Ninety-nine genes comprising various interleukin classes with NES of −3.00 were affected. All the downregulated genes are coloured in green boxes. Upstream genes in the two major chemokines subfamilies of CC and CXC are affected. The image was generated using KEGG Mapper (copyright permission obtained from Kanehisa Laboratory) [27].

In this study, NES indicates whether the gene set is enriched in AML-NK (upregulated or downregulated) and the magnitude of the enrichment. The blue bars indicate positive NES (upregulation), while the orange bars indicate negative enrichment (downregulation). FDR of 0.05 shows that, on average, 5% of the significant results obtained in this study are likely to be false positives. The darker shades of the bar graph indicate FDR ≤ 0.05, while the lighter shade indicates FDR ≥ 0.05. Most significantly enriched pathways are summarised in S IX. Cell cycle (hsa 04110) was the most positively enriched significant pathway, whereas cytokine-cytokine receptor interaction (hsa04060) was the most negatively enriched significant pathway in the GSEA analysis of AML-NK patients versus the healthy controls. Fig 2 illustrates the cell cycle (hsa04110) and 56 genes upregulated (red boxes) involved all four cell cycle phases, including G1-phase, S-phase, G2-phase, and M-phase, as shown. Cytokine-cytokine receptor interaction (hsa04060) downregulated genes (green boxes) are shown in Fig 3.

An in-depth clustering of pathways unveiled the top 19 most significantly enriched pathways and relevant to AML-NK pathogenesis consisted of four major overlapping categories as follows: major histocompatibility (MHC) antigen presentation pathways (n = 6), cell signalling pathways (n = 11), DNA replication and cell cycle (n = 2) and haematopoietic cell lineage pathway (n = 1).In the MHC antigen presentations, the following pathways were involved: allograft rejection (hsa05330), type I diabetes mellitus (hsa04940), antigen processing and presentation (hsa04612), natural killer cell-mediated cytotoxicity (hsa04650), autoimmune thyroid disease (hsa05320), and graft-versus-host disease (hsa05332). Various signalling pathways were affected in the eleven signalling pathways category as follows: MAPK signalling pathway (cell cycle (hsa04110), JAK-STAT signalling pathway (hsa04630), T-cell receptor signalling pathway (hsa04660), Th1 and Th2 cell differentiation (hsa04658), Th17 cell differentiation (hsa04659), and osteoclast differentiation (hsa04380)), JAK-STAT signalling pathway (chemokine signalling pathway (hsa04062), Th17 cell differentiation (hsa04659), inflammatory bowel disease (IBD) (hsa05321) and osteoclast differentiation (hsa04380), NF-κB signalling pathway (Th1 and Th2 cell differentiation (hsa04658), NF-kappa B signalling pathway (hsa04064), TGF-β pathway (endocytosis (hsa04144), inflammatory bowel disease (IBD) (hsa05321) and Th17 cell differentiation (hsa04659), PI3K-Akt pathway (osteoclast differentiation (hsa04380), T-cell receptor signalling pathway (hsa04660), and JAK-STAT signalling pathway (hsa04630), and cytokine-cytokine receptor interaction (hsa04060). Apart from MHC antigen presentation pathways and signalling pathways, DNA replication (hsa03030) that impacts the cell cycle pathway (hsa04110) was also enriched in this study. The haematopoietic cell lineage (hsa04640) was also among the significantly enriched pathways in the GSEA analysis.

### AML-NK DX versus paired CR1 sample groups

**Fig 4:**
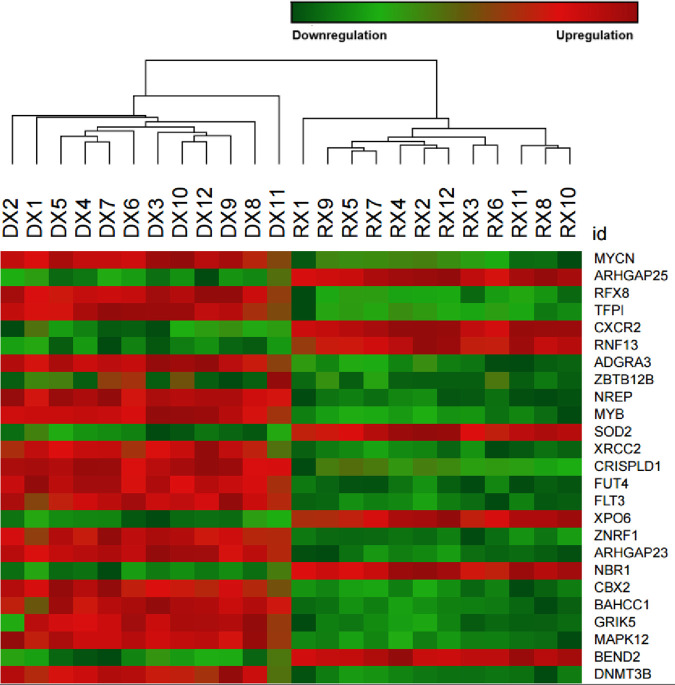
Hierarchical clustering heatmap of AML-NK DX and CR1 samples. The heat map depicts the correlations between the DX and their CR1 samples by the colour-coded gradient between green, indicating downregulation, and red, indicating upregulation.

Unsupervised clustering was conducted on 12 pairs of AML-NK (DX and CR1), and the findings are depicted in a PCA plot, volcano plot, and hierarchical clustering heatmap, available in S X. This study discovered 5,621 dysregulated genes, encompassing 4,761 (84.7%) coding genes and 860 (15.3%) non-coding elements after filtering based on the following: p-adj < 0.01, log2FoldChange >1 and <−1, and BaseMean > 100. About 3,031 (53.9%) of these genes were downregulated, and 2,590 (46.1%) were upregulated in the AML-NK (DX) in comparison to their paired CR1 sample. A comprehensive literature search on this cohort’s top 100 significant DEGs disclosed that 25.0% (n = 25/100) of these DEGs were reported in other AML studies (last update date of 19^th^ April 2025), as summarised in S XI. The regulations of the 25 DEGs in this cohort were consistent with the findings from other AML studies, as summarised in Table 1. The hierarchical clustering heatmap of these 25 DEGs is shown in Fig 4.

**Table 1:** DEG findings of the 25 genes reported in AM-NK in this study were consistent with other AML-related published literature.

Gene symbol	log2FC	padj	Gene role	Regulation information in other studies
*NREP*	3.91	1.07E-121	Unknown	Upregulated [28]
*ARHGAP23*	4.43	5.59E-104	Unknown	Upregulated [29]
*FUT4*	3.04	5.59E-104	Unknown	Upregulated [30]
*MAPK12*	5.19	5.29E-79	Unknown	Upregulated [31]
*FLT3*	4.88	3.78E-77	Oncogene	Upregulated [32]
*MYB*	5.14	2.62E-76	Oncogene	Upregulated [32]
*BAHCC1*	4.96	1.08E-72	Unknown	Upregulated [33]
*BEND2*	−6.99	3.07E-71	Unknown	Downregulated [34]
*ZNRF1*	3.2	8.63E-66	Unknown	Upregulated [35]
*CRISPLD1*	5.68	2.66E-62	Unknown	Upregulated [36]
*RFX8*	6.15	3.01E-62	Unknown	Upregulated [37]
*CXCR2*	−7.31	3.34E-59	Unknown	Downregulated [38]
*DNMT3B*	5.34	4.36E-56	Oncogene	Upregulated [39]
*ARHGAP25*	−2.91	4.70E-55	Unknown	Downregulated [40]
*GRIK5*	5.46	4.90E-51	Unknown	Upregulated [41]
*CBX2*	4.76	1.83E-49	Unknown	Upregulated [42]
*RNF13*	−2.61	3.94E-49	Unknown	Downregulated [43]
*SOD2*	−4.33	6.16E-49	Unknown	Downregulated [44]
*ZBTB12*	3.1	5.18E-48	Unknown	Upregulated [45]
*XPO6*	−3.35	2.13E-48	Unknown	Downregulated [46]
*ADGRA3*	4.36	9.07E-48	Unknown	Upregulated [47]
*NBR1*	−1.77	1.35E-47	Unknown	Downregulated [48]
*TFPI*	5.8	2.20E-47	Unknown	Upregulated [49]
*XRCC2*	3.22	2.19E-45	Unknown	Upregulated [50]
*MYCN*	6.5	2.38E-38	Oncogene	Upregulated [51]

FC refers to fold change; padj refers to the adjusted p-value.

### Functional enrichment analyses (PA) using the GSEA function on the WebGestalt for DEGs in AML-NK Dx versus paired CR1 sample groups comparison

**Fig 5:**
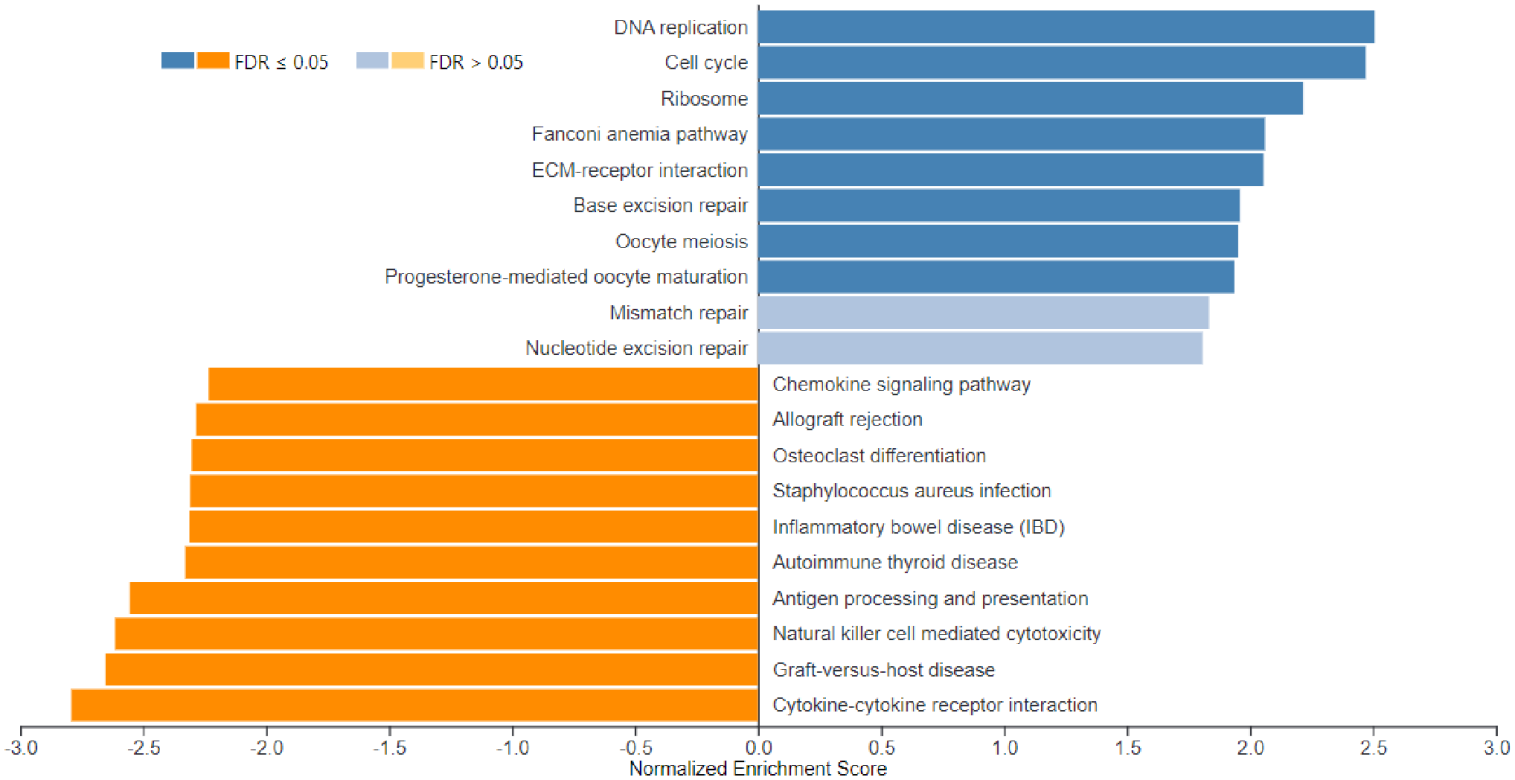
Bar chart depicting significantly enriched pathways in the GSEA analysis of AML-NK DX and CR1 samples. The bar width equals the enrichment ratio, with blue indicating positively related categories and orange indicating negatively related categories in GSEA analysis. Ten categories were positively enriched, of which eight had a p-value, FDR below 0.05, and two other pathways (mismatch repair and nucleotide excision repair) had a p-value of below 0.05 and FDR above 0.05. Ten pathways had a p-value and FDR below 0.05 in the negatively enriched category. This figure was generated using the WebGestalt tool [1].

Functional enrichment analysis (PA) using the GSEA function on the WebGestalt tool was performed using the 5621 dysregulated genes, which discovered that 20 categories of pathways were affected, as shown in Fig 5. In this study, NES indicates whether the gene set is enriched in AML-NK (upregulated or downregulated) and the magnitude of the enrichment. The blue bars indicate positive NES (upregulation), while the orange bars indicate negative enrichment (downregulation). FDR of 0.05 shows that, on average, 5% of the significant results obtained in this study are likely to be false positive. The darker shades of the bar graph indicate FDR ≤ 0.05, while the lighter shade indicates FDR ≥ 0.05.

**Fig 6:**
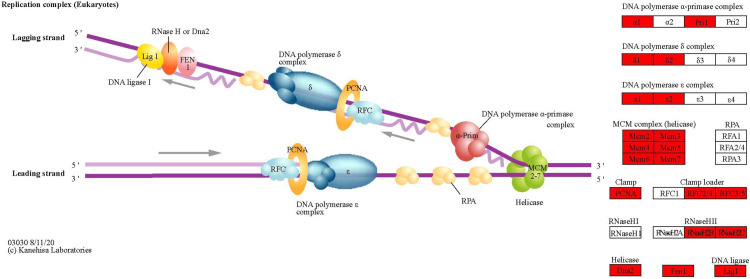
DNA replication (hsa03030) pathway. Cytokine-cytokine receptor interaction (hsa04060) was the most negatively enriched significant pathway in this cohort’s GSEA analysis of 12 paired DX and CR1 AML-NK patients (not shown). Twenty genes were upregulated with NES of 2.5076. All the upregulated genes (red boxes) are involved in the DNA replication.Upstream genes of DNA polymerase α, δ and ε involved in nuclear DNA replication were upregulated. The upstream genes in the MCM complex that controls the cell cycle DNA replication were also upregulated, as shown in red boxes. DNA2, FEN1, and DNA ligase (Lig1) essential for DNA replication were upregulated. The image was generated using KEGG Mapper (copyright permission obtained from Kanehisa Laboratories) (Kanehisa, 2000). This figure was generated using the WebGestalt tool [24].

DNA replication (hsa03030) was the most positively enriched significant pathway, as shown in Fig 6. An in-depth clustering of pathways unveiled the top 15 most significantly enriched pathways, which consisted of four major overlapping categories as follows: major histocompatibility (MHC) antigen presentation pathways (n = 5), cell signalling pathways (n = 4), DNA replication and cell cycle (n = 3) and other miscellaneous pathways (n = 2). In the MHC antigen presentation, the following pathways were affected: allograft rejection (hsa05330), antigen processing and presentation (hsa04612), autoimmune thyroid disease (hsa05320), natural killer cell-mediated cytotoxicity (hsa04650), and graft-versus-host disease (hsa05332). Cytokine-cytokine receptor interaction was the most negatively enriched pathway. Several signalling pathways were also enriched in this as follows: MAPK signalling pathway (oocyte meiosis (hsa04114), progesterone mediated oocyte maturation (hsa04914), Osteoclast differentiation (hsa04380) and cell cycle (hsa04110), PI3K-Akt, NF-kB and JAK-STAT signalling pathways in Osteoclast differentiation (hsa04380), TGF-β and JAK-STAT signalling pathway in Inflammatory bowel disease (hsa05321) and Chemokine signalling pathway (hsa04062). Three pathways that were related to DNA replication and cell cycle processes were positively enriched, including DNA replication (hsa03030), cell cycle (hsa04110), and base excision repair (hsa03410).

### qPCR validation for DEGs

Reverse transcription-quantitative real-time PCR (RT-qPCR) was performed on the six most significant not reported in AML cases in the DEG profile of AML-NK versus healthy controls. The findings of these DEGs, as listed in Table 2 by transcriptome sequencing, concorded with the RT-qPCR findings. The RT-qPCR findings are described in S XII.

**Table 2:** Validation of DEG by RT-qPCR.

Gene	Transcriptome sequencing		RT-qPCR		Regulation
	log2FC	padj	FC(2^-∆∆Ct^)	p-value	
*TDP2*	−2.31	9.6E-124	0.4	0.00017	Downregulated
*STK40*	−2.55	3.17E-81	0.1	0.00009	Downregulated
*PACS1*	−1.77	2.86E-74	0.2	0.00036	Downregulated
*OAZ2*	−2.29	1.04E-73	0.2	0.00009	Downregulated
*TCF20*	−1.59	1.97E-67	0.2	0.00041	Downregulated
*CRISPLD1*	6.27	1.36E-65	383.0	6E-08	Upregulated

FC refers to fold change. FC(2^-∆∆Ct^) for the controls in RT-qPCR was 1 for the six genes. Regulation data was generated based on the FC(2^-∆∆Ct^) between the controls and the sample.

## Discussion

In this study, in-depth DEG profiling of AML-NK was performed by conducting several subgroup analyses, including comparison against the healthy control groups and evaluation of DX and CR1 AML-NK patients. Next, the DEG profiles for each subgroup were assessed for dysregulated pathways and exemplified significantly affected pathways implicated in AML-NK pathogenesis. Unsupervised clustering and DEG analysis between the AML-NK patients and healthy controls disclosed 5126 DEGs that included the coding (85.8%) and non-coding elements (14.2%) affecting 37 categories of pathways, indicating widespread transcriptomic dysregulation, suggesting disrupted haematopoietic processes and immune responses. These findings align with the known AML pathobiology, which states that arrest in cell differentiation and proliferation are hallmarks of this disease [52].

This study is the first to summarise the comprehensive literature search of the top 600 DEGs in the AML-NK versus healthy control that included information on the novelty of the findings, fold change of each DEG, the role of the gene (oncogene/TSG), type (coding/non-coding genes), evidence in AML (reported in AML or novel in AML) and in other cancers/diseases. This study’s literature search disclosed that 15.8% of the DEGs were novel to AML-NK, reinforcing the unique features of AML-NK that require subtype-specific therapeutic strategies. Interestingly, 12.2% (n = 73/600) of the dysregulated non-coding elements in this study comprise lncRNAs, lincRNAs, and miRNAs. Although historically, the identification of coding genes was of interest as potential biomarkers in AML studies, more recently, the initially considered “dark matter” non-coding genes are also crucial in synchronising the gene expressions at various processes, including proliferation, survival, differentiation, and genomic stability [53–56]. Several studies have reported similar findings in the significantly enriched pathways described in this study [57,58].

The most positively enriched pathway (cell cycle, hsa04110) revealed several genes were upregulated in all the cell cycle phases (G1, S, G2, and M phase), contributing to increased proliferation that predisposes to genomic instability in these AML-NK patients [59]. These upregulated genes in the cell cycle pathway also alter the proliferative regulators, which may result in the uncontrolled proliferation of abnormal cells during leukaemogenesis [60]. The most negatively enriched significant pathway (cytokine-cytokine receptor interaction (hsa04060) is reported to be dysregulated in all forms of leukaemia, where these cytokines function in contrast to their normal function by serving a critical part of the malignancy program, including regulating the differentiation and growth of malignant cells [61]. The marked downregulation of genes in the cytokine-cytokine receptor interaction suggests impaired immune response and cell communication, allowing leukemic cell evasion from immune surveillance, hence causing disease progression. Dysregulated immune-related pathways such as antigen processing and presentation and NK cell-mediated cytotoxicity suggest an altered immunological milieu in AML-NK, which may contribute to immune escape and disease persistence [62].

A comprehensive clustering of pathways revealed the 19 most significantly enriched pathways, categorised into four primary overlapping groups: major histocompatibility (MHC) antigen presentation pathways (n = 6), cell signalling pathways (n = 11), DNA replication and cell cycle (n = 2), and haematopoietic cell lineage pathway (n = 1)An in-depth literature review revealed that all ten signalling pathways have been reported in AML and other types of cancers, with none specific to the AML-NK cohort in this study [63–66]. The genes implicated in the MHC antigen pathways categories were downregulated in line with reports on the hallmarks of cancer. As the MHC-I and MHC-II are vital components of the antigen presentation machinery accountable for presenting neoantigen to CD8+ and CD4 + T lymphocytes, respectively, the downregulation leads to immune evasion deployed by tumour cells [67,68]. Downregulation of the MHC-I, which is associated with a dismal prognosis, has been described in four to 90% of human tumours [67].

In the DEG profiling of AML-NK patients, about 72% (n = 18/25) of the genes that were reported in AML studies were overexpressed in the DX samples that included several oncogenes (*FLT3, MYB, DNMT3B,* and *MYCN*). Studies have reported that *FLT3* overexpression is observed in about 93% of AML patients [69,70]. Other studies have reported the overexpression of *MYB, DNMT3B*, and *MYCN* in acute myeloid leukaemia patients [51,71–73]. The *FLT3* and *MYCN* genes are critical in the transcriptional misregulation pathway in cancer, with *FLT3* functioning in signal mediation. At the same time, *MYCN* is involved in cell apoptosis, self-renewal, and migration. As there are only limited MRD monitoring markers for the AML-NK patients, such as *NPM1* and *FLT3*-ITD mutations, overexpression of the oncogenes (*FLT3, MYB, DNMT3B,* and *MYCN*) can be useful in MRD monitoring, especially in cases with no genetic aberrations. It will be crucial to select the most significantly overexpressed gene to create an MRD monitoring system, possibly supplemented by other technologies, such as multiparametric flow cytometry in cases of AML-NK with no genetic markers at diagnosis [74].

As the dysregulation of these genes is evident in the DX patients against their paired CR1 samples, these DEGs exhibit potential utility for MRD monitoring of AML-NK patients. The upregulation of *FLT3, MYB, DNMT3B*, and *MYCN* oncogenes was also seen in the DX and paired CR1 DEG profiling, reiterating their potentiality for MRD monitoring in AML-NK patients. Other studies have suggested that gene expression is a suitable marker for MRD monitoring, especially in AML-NK patients who lack genetic mutations. [75,76].

The consistent upregulation of the DNA replication (hsa03030) pathway across AML-NK vs healthy controls and DX vs CR1 comparisons underscores the central role of proliferative dysregulation in AML-NK pathogenesis. These pathways involve key replisome machinery components, including MCM complex proteins, DNA polymerases and replication initiation factors. Overexpression of these genes contributes to unchecked DNA synthesis and cell cycle progression that accelerates clonal expansion of leukaemia blast [77]. The consistent upregulation of the DNA replication (hsa03030) pathway across AML-NK vs healthy controls and DX vs CR1 comparisons underscores the central role of proliferative dysregulation in AML-NK pathogenesis. These pathways involve key replisome machinery components, including MCM complex proteins, DNA polymerases and replication initiation factors. Overexpression of these genes contributes to unchecked DNA synthesis and cell cycle progression that accelerate clonal expansion of leukaemia blasts. Abnormal activation of replication machinery facilitates leukemic cell proliferation and increases replication stress, a hallmark of cancer, leading to genomic instability and the acquisition of additional mutations. Preclinical studies on AML models targeting MCM helicases and CDC7 kinase by small molecule inhibitors essential for replication initiation support this study’s findings, highlighting dysregulation of DNA replication pathway [78]. Moreover, the persistence of DNA replication pathway upregulation during DX compared to CR1 has the potential for MRD assessment at the molecular level, including in cases where morphological full remission has been achieved [76,79].

The downregulation of the cytokine-cytokine receptor interaction (hsa04060) pathway across all comparisons revealed significant suppression of immune communication networks in AML-NK. This pathway encompasses an array of interleukins, chemokines, interferons, and their receptors, which are crucial for the activation, survival, and trafficking of immune cells. Downregulation of this pathway leads to an impaired immune environment in the bone marrow niche, possibly facilitating immune evasion in leukemic cells. The immune suppression affects innate immune responses, including NK cell and macrophage activity and adaptive immune surveillance mechanisms mediated by T and B lymphocytes. As reported by other researchers, the reduced expression of key cytokines and their receptors possibly contributed to the dysfunction of the T-cell and NK-cell compartments in AML patients [80,81].

Furthermore, cytokine signalling is crucial in normal haematopoiesis. This finding may substantiate the justification for cytokine-based immunotherapies or immune checkpoint inhibition in AML-NK. Restoring immunological signalling via cytokine supplementation (e.g., IL-15 super agonists) or augmenting antigen presentation to synergise with conventional chemotherapy or novel targeted therapies to enhance immune-mediated disease management [82,83]

Dysregulation of proliferative pathways, such as MAPK, NF-kappa B signalling pathway PI3K-AKT, and JAK-STAT signalling, emphasises the central role of uncontrolled cell proliferation and survival in AML development [83,84]. Aberrant activation of these signalling pathways facilitates leukemic blast proliferation, increases resistance to apoptosis, and exacerbates disease progression [83,84]. These findings in this study aligned with previous research highlighting the critical role of proliferative signalling in leukemogenesis [83,84].In contrast, pathways related to immunological function, such as antigen processing and presentation, T-cell receptor signalling, and interferon-mediated responses, were downregulated, indicating significant immune suppression [85,86]. AML cells elude immune monitoring by downregulating MHC molecules and secreting immunosuppressive cytokines, which limit T-cell activation and foster an immunosuppressive milieu [87]. This immunological malfunction promotes disease persistence and resistance. The invasion of AML cells into immune surveillance through the downregulation of MHC molecules and the secretion of immunosuppressive cytokines, which inhibit T-cell activation and promote an immunosuppressive microenvironment, is a known mechanism [87]. This immune dysfunction sustains disease persistence and therapeutic resistance.

The complex, dichotomous nature of AML pathogenesis is underscored by the simultaneous activation of proliferative pathways and suppression of immune responses, which result in the rapid expansion of leukemic clones and the evasion of host immunological defences. This discovery is consistent with the current therapeutic paradigm, which advocates using combined methods that address the proliferative apparatus and the immune-suppressive mechanisms of AML [88,89]. By targeting these opposing yet interrelated mechanisms, it may be possible to enhance the therapeutic effectiveness and clinical outcomes of AML-NK patients.

## Conclusion

This study presents a comprehensive transcriptomic analysis of AML-NK, revealing key dysregulations in proliferative and immune-related pathways. Upregulation of the cell cycle and DNA replication pathways highlights uncontrolled proliferation, while downregulation of cytokine signalling and MHC antigen presentation suggests impaired immune surveillance. These findings reflect the dual mechanisms of leukemogenesis—enhanced proliferation and immune evasion. We identified novel DEGs, including non-coding RNAs, expanding the landscape of potential AML-NK biomarkers. The validation of unreported genes via RT-qPCR affirms the robustness of the transcriptomic data. Additionally, consistent overexpression of oncogenes such as *FLT3*, *MYB*, *DNMT3B*, and *MYCN* supports their potential role in MRD monitoring, especially in patients without genetic aberrations. Overall, our results underscore the unique molecular profile of AML-NK and advocate for combined therapeutic strategies targeting proliferative and immunosuppressive pathways to improve clinical outcomes.

## Supporting information

S1 File Sample size calculation. (DOCX)

S2 File Subject inclusion and exclusion criteria. (DOCX)

S3 File DEG Pipeline. (DOCX)

S4 File The RT-qPCR protocols. (DOCX)

S5 File Patient demographic and clinical characteristics. (DOCX)

S6 File Quality control data of transcriptome sequencing. (DOCX)

S7 File The principal component analysis (PCA), volcano plot and a hierarchical clustering heatmap of AML-NK vs healthy controls. (DOCX)

S8 File Top 600 DEGs in AML-NK vs healthy controls. (XLSX)

S9 File Most significantly enriched pathways in AML-NK (DX-CR1). (DOCX)

S10 File The principal component analysis (PCA), volcano plot and a hierarchical clustering heatmap of AML-NK (DX-CR1). (DOCX)

S11 File Top 100 significant DEGS between AML-NK (DX-CR1). (DOCX)

S12 File The RT-qPCR findings of AML-NK vs healthy controls DEGs. (DOCX)

## References

[1] Döhner H, Weisdorf DJ, Bloomfield CD. Acute Myeloid Leukemia. N Engl J Med. 2015;373(12):1136–52. doi: 10.1056/NEJMra1406184 26376137

[2] Mawad R, Estey EH. Acute myeloid leukemia with normal cytogenetics. Curr Oncol Rep. 2012;14(5):359–68. doi: 10.1007/s11912-012-0252-x 22806102

[3] Grimwade D, Haferlach T. Gene-expression profiling in acute myeloid leukemia. N Engl J Med. 2004;350(16):1676–8. doi: 10.1056/NEJMe048040 15084701

[4] Grimwade D, Hills RK. Independent prognostic factors for AML outcome. Hematol Am Soc Hematol Educ Program. 2009;:385–95. doi: 10.1182/asheducation-2009.1.385 20008224

[5] Medinger M, Passweg JR. Acute myeloid leukaemia genomics. Br J Haematol. 2017;179(4):530–42. doi: 10.1111/bjh.14823 28653397

[6] Short NJ, Rytting ME, Cortes JE. Acute myeloid leukaemia. Lancet. 2018;392(10147):593–606. doi: 10.1016/S0140-6736(18)31041-9 30078459 PMC10230947

[7] Roychowdhury S, Chinnaiyan AM. Translating cancer genomes and transcriptomes for precision oncology. CA Cancer J Clin. 2016;66(1):75–88. doi: 10.3322/caac.21329 26528881 PMC4713245

[8] Fan Y, Hu Y, Nguyen C, Yan C, Ries RE, Bolouri H. Altered transcriptome uniquely associated with AML vs. normal hematopoiesis. 2017. 10.1182/blood.V130.Suppl_1.3792.3792

[9] Bullinger L, Döhner K, Bair E, Fröhling S, Schlenk RF, Tibshirani R, et al. Use of gene-expression profiling to identify prognostic subclasses in adult acute myeloid leukemia. N Engl J Med. 2004;350(16):1605–16. doi: 10.1056/NEJMoa031046 15084693

[10] Foran JM. New prognostic markers in acute myeloid leukemia: perspective from the clinic. Hematology Am Soc Hematol Educ Program. 2010;2010:47–55. doi: 10.1182/asheducation-2010.1.47 21239770

[11] Ye J, Luo D, Yu J, Zhu S. Transcriptome analysis identifies key regulators and networks in Acute myeloid leukemia. Hematology. 2019;24(1):487–91. doi: 10.1080/16078454.2019.1631506 31210592

[12] Shiba N, Yoshida K, Shiraishi Y, Yuichi S, Hara Y, Yamato G, et al. Transcriptome Analysis Revealed the Entire Genetic Understanding of Pediatric Acute Myeloid Leukemia with a Normal Karyotype. Blood. 2016;128(22):2850–2850. doi: 10.1182/blood.v128.22.2850.2850

[13] Maiga A, Lemieux S, Pabst C, Lavallée V-P, Bouvier M, Sauvageau G, et al. Transcriptome Analysis Reveals That G Protein-Coupled Receptors Are Potential Diagnostic Markers or Therapeutic Targets in Acute Myeloid Leukemia. Blood. 2015;126(23):3855–3855. doi: 10.1182/blood.v126.23.3855.3855

[14] Wang J, Uddin MN, Hao J-P, Chen R, Xiang Y-X, Xiong D-Q, et al. Identification of Potential Novel Prognosis-Related Genes Through Transcriptome Sequencing, Bioinformatics Analysis, and Clinical Validation in Acute Myeloid Leukemia. Front Genet. 2021;12:723001. doi: 10.3389/fgene.2021.723001 34777462 PMC8585857

[15] Wu A-Y, Yang H-C, Lin C-M, Wu B, Qu Q-S, Zheng Y-H, et al. The Transcriptome Study of Subtype M2 Acute Myeloblastic Leukemia. Cell Biochem Biophys. 2015;72(3):653–6. doi: 10.1007/s12013-014-0432-4 27352183

[16] Osman SH, Abu N, Aziz H, Chow YP, Wan Mohamad Nazarie WF, Ab Mutalib N-S, et al. Deep Transcriptome Sequencing of Pediatric Acute Myeloid Leukemia Patients at Diagnosis, Remission and Relapse: Experience in 3 Malaysian Children in a Single Center Study. Front Genet. 2020;11:66. doi: 10.3389/fgene.2020.00066 32174960 PMC7056821

[17] Zhang B, Kirov S, Snoddy J. WebGestalt: an integrated system for exploring gene sets in various biological contexts. Nucleic Acids Res. 2005;33(Web Server issue):W741-8. doi: 10.1093/nar/gki475 15980575 PMC1160236

[18] Chang KM, Guan YK, Jameela S, Jay S, Kuan JW, Lau NS. Ampang Protocol. 2012th ed. Haematology Department. 2012.

[19] Ganapule A, Nemani S, Korula A, Lakshmi KM, Abraham A, Srivastava A, et al. Allogeneic Stem Cell Transplant for Acute Myeloid Leukemia: Evolution of an Effective Strategy in India. J Glob Oncol. 2017;3(6):773–81. doi: 10.1200/JGO.2016.006650 29244983 PMC5735966

[20] Schurch NJ, Schofield P, Gierliński M, Cole C, Sherstnev A, Singh V, et al. How many biological replicates are needed in an RNA-seq experiment and which differential expression tool should you use?. RNA. 2016;22(6):839–51. doi: 10.1261/rna.053959.115 27022035 PMC4878611

[21] Liao Y, Smyth GK, Shi W. featureCounts: an efficient general purpose program for assigning sequence reads to genomic features. Bioinformatics. 2014;30(7):923–30. doi: 10.1093/bioinformatics/btt656 24227677

[22] Love MI, Huber W, Anders S. Moderated estimation of fold change and dispersion for RNA-seq data with DESeq2. Genome Biol. 2014;15(12):550. doi: 10.1186/s13059-014-0550-8 25516281 PMC4302049

[23] Wu H, Wang C, Wu Z. A new shrinkage estimator for dispersion improves differential expression detection in RNA-seq data. Biostatistics. 2013;14(2):232–43. doi: 10.1093/biostatistics/kxs033 23001152 PMC3590927

[24] Liao Y, Wang J, Jaehnig EJ, Shi Z, Zhang B. WebGestalt 2019: gene set analysis toolkit with revamped UIs and APIs. Nucleic Acids Res. 2019;47(W1):W199–205. doi: 10.1093/nar/gkz401 31114916 PMC6602449

[25] Chen S, Zhou Y, Chen Y, Gu J. fastp: an ultra-fast all-in-one FASTQ preprocessor. Bioinformatics. 2018;34(17):i884–90. doi: 10.1093/bioinformatics/bty560 30423086 PMC6129281

[26] Dobin A, Davis CA, Schlesinger F, Drenkow J, Zaleski C, Jha S, et al. STAR: ultrafast universal RNA-seq aligner. Bioinformatics. 2013;29(1):15–21. doi: 10.1093/bioinformatics/bts635 23104886 PMC3530905

[27] Kanehisa M, Goto S. KEGG: kyoto encyclopedia of genes and genomes. Nucleic Acids Res. 2000;28(1):27–30. doi: 10.1093/nar/28.1.27 10592173 PMC102409

[28] Thomas BE, Perumalla P, Bhasin SS, Sarkar D, Dwivedi B, Park SI, et al. Single Cell Transcriptomics Revealed AML and Non-AML Cell Clusters Relevant to Relapse and Remission in Pediatric AML. Blood. 2020;136(Supplement 1):24–5. doi: 10.1182/blood-2020-14251332430494

[29] Minařík L, Pimková K, Kokavec J, Schaffartziková A, Vellieux F, Kulvait V, et al. Analysis of 5-Azacytidine Resistance Models Reveals a Set of Targetable Pathways. Cells. 2022;11(2):223. doi: 10.3390/cells11020223 35053339 PMC8774143

[30] Dai X, Shen L. Advances and Trends in Omics Technology Development. Front Med (Lausanne). 2022;9:911861. doi: 10.3389/fmed.2022.911861 35860739 PMC9289742

[31] Wang J, Song Z, Ren L, Zhang B, Zhang Y, Yang X, et al. Pan-cancer analysis supports MAPK12 as a potential prognostic and immunotherapeutic target in multiple tumor types, including in THCA. Oncol Lett. 2022;24(6):445. doi: 10.3892/ol.2022.13565 36420075 PMC9647794

[32] Cheng J, Qu L, Wang J, Cheng L, Wang Y. High expression of FLT3 is a risk factor in leukemia. Mol Med Rep. 2018;17(2):2885–92. doi: 10.3892/mmr.2017.8232 29257272 PMC5783504

[33] Fan H, Lu J, Guo Y, Li D, Zhang Z-M, Tsai Y-H, et al. BAHCC1 binds H3K27me3 via a conserved BAH module to mediate gene silencing and oncogenesis. Nat Genet. 2020;52(12):1384–96. doi: 10.1038/s41588-020-00729-3 33139953 PMC8330957

[34] Lauber C, Correia N, Trumpp A, Rieger MA, Dolnik A, Bullinger L, et al. Survival differences and associated molecular signatures of DNMT3A-mutant acute myeloid leukemia patients. Sci Rep. 2020;10(1):12761. doi: 10.1038/s41598-020-69691-8 32728112 PMC7391693

[35] Chen P, Liu Y, Zhang R, Wang H, Zhang J, Guo M, et al. Adaptive immunity-related gene expression profile is correlated with clinical phenotype in patients with acute myeloid leukemia. Ann Transl Med. 2021;9(11):939. doi: 10.21037/atm-21-2720 34350254 PMC8263877

[36] Zhuang H, Chen Y, Sheng X, Hong L, Gao R, Zhuang X. Searching for a signature involving 10 genes to predict the survival of patients with acute myelocytic leukemia through a combined multi-omics analysis. PeerJ. 2020;8:e9437. doi: 10.7717/peerj.9437 32617195 PMC7321666

[37] Alom MM, Faruqe MO, Molla MKI, Rahman MM. Exploring Prognostic Biomarkers of Acute Myeloid Leukemia to Determine Its Most Effective Drugs from the FDA-Approved List through Molecular Docking and Dynamic Simulation. Biomed Res Int. 2023;2023:1946703. doi: 10.1155/2023/1946703 37359050 PMC10287530

[38] Tang W, Li Z, Li X, Huo Z. High CXCR2 expression predicts poor prognosis in adult patients with acute myeloid leukemia. Ther Adv Hematol. 2020;11:2040620720958586. doi: 10.1177/2040620720958586 32973988 PMC7493249

[39] Bhogal B, Weir BA, Crescenzo R, Marien A, Kwon MC, Philippar U, et al. The methyltransferase domain of DNMT1 is an essential domain in acute myeloid leukemia independent of DNMT3A mutation. Commun Biol. 2022;5(1):1174. doi: 10.1038/s42003-022-04139-5 36329185 PMC9633652

[40] Han C, He S, Wang R, Gao X, Wang H, Qiao J, et al. The role of ARHGAP9: clinical implication and potential function in acute myeloid leukemia. J Transl Med. 2021;19(1):65. doi: 10.1186/s12967-021-02733-5 33579308 PMC7881617

[41] Xia J, Garg S, He L, Jagdhane P, Carsten M‐T., Caroline P. PS980 GRIK5 drives self renewal pathways in human acute myeloid LEUKEMIA CELLS. HemaSphere. 2019;3(S1):440–1. doi: 10.1097/01.hs9.0000562220.27971.d0

[42] Ha CJ, Hu W, Elias HK, Chakraborty S, Park CY. Mir-29 Maintains the Acute Myeloid Leukemia Epigenome By Regulating CBX2. Blood. 2019;134(Supplement_1):1236–1236. doi: 10.1182/blood-2019-131518

[43] Zhang R, Li Y, Wang H, Zhu K, Zhang G. The Regulation of circRNA RNF13/miRNA-1224-5p Axis Promotes the Malignant Evolution in Acute Myeloid Leukemia. Biomed Res Int. 2020;2020:5654380. doi: 10.1155/2020/5654380 33083473 PMC7557902

[44] Sillar JR, Germon ZP, DeIuliis GN, Dun MD. The Role of Reactive Oxygen Species in Acute Myeloid Leukaemia. Int J Mol Sci. 2019;20(23):6003. doi: 10.3390/ijms20236003 31795243 PMC6929020

[45] Kuang Y, Wang Y, Cao X, Peng C, Gao H. New prognostic factors and scoring system for patients with acute myeloid leukemia. Oncol Lett. 2021;22(6):823. doi: 10.3892/ol.2021.13084 34691250 PMC8527825

[46] Luo H, Zhang Y, Hu N, He Y, He C. Systematic Construction and Validation of an RNA-Binding Protein-Associated Prognostic Model for Acute Myeloid Leukemia. Front Genet. 2021;12:715840. doi: 10.3389/fgene.2021.715840 34630514 PMC8498117

[47] Fu J-F, Wen C-J, Yen T-H, Shih L-Y. Hoxa11-mediated reduction of cell migration contributes to myeloid sarcoma formation induced by cooperation of MLL/AF10 with activating KRAS mutation in a mouse transplantation model: Hoxa11 in myeloid sarcoma formation. Neoplasia. 2022;29:100802. doi: 10.1016/j.neo.2022.100802 35500545 PMC9065885

[48] Liang PQ, Miao M, Liu ZG, Hu R, Jiang HN, Li C, et al. Expression of autophagy genes in acute myeloid leukemia: associations with clinical characteristics and prognosis. Neoplasma. 2018;65(5):807–14. doi: 10.4149/neo_2018_171028N691 29940753

[49] Cui XY, Tjønnfjord GE, Kanse SM, Dahm AEA, Iversen N, Myklebust CF, et al. Tissue factor pathway inhibitor upregulates CXCR7 expression and enhances CXCL12-mediated migration in chronic lymphocytic leukemia. Sci Rep. 2021;11(1):5127. doi: 10.1038/s41598-021-84695-8 33664415 PMC7933411

[50] Padella A, Hutter S, Walter W, Baer C, Azzali I, et al. In: Cancer Research, Philadelphia (PA), 2022.

[51] Liu L, Xu F, Chang C-K, He Q, Wu L-Y, Zhang Z, et al. MYCN contributes to the malignant characteristics of erythroleukemia through EZH2-mediated epigenetic repression of p21. Cell Death Dis. 2017;8(10):e3126. doi: 10.1038/cddis.2017.526 29022893 PMC5682688

[52] Papaemmanuil E, Gerstung M, Bullinger L, Gaidzik VI, Paschka P, Roberts ND, et al. Genomic classification and prognosis in acute myeloid leukemia. N Engl J Med. 2016;374(23):2209–21. doi: 10.1056/NEJMoa1516192 27276561 PMC4979995

[53] Gourvest M, Brousset P, Bousquet M. Long Noncoding RNAs in Acute Myeloid Leukemia: Functional Characterization and Clinical Relevance. Cancers (Basel). 2019;11(11):1638. doi: 10.3390/cancers11111638 31653018 PMC6896193

[54] Hung T, Wang Y, Lin MF, Koegel AK, Kotake Y, Grant GD, et al. Extensive and coordinated transcription of noncoding RNAs within cell-cycle promoters. Nat Genet. 2011;43(7):621–9. doi: 10.1038/ng.848 21642992 PMC3652667

[55] Mercer TR, Dinger ME, Mattick JS. Long non-coding RNAs: insights into functions. Nat Rev Genet. 2009;10(3):155–9. doi: 10.1038/nrg2521 19188922

[56] Geisler S, Coller J. RNA in unexpected places: long non-coding RNA functions in diverse cellular contexts. Nat Rev Mol Cell Biol. 2013;14(11):699–712. doi: 10.1038/nrm3679 24105322 PMC4852478

[57] Fu W, Cheng G, Ding Y, Deng Y, Guo P. Identification of hub genes and its correlation with the prognosis of acute myeloid leukemia based on high‐throughput data analysis. Precision Radiat Oncol. 2020;4(2):49–56. doi: 10.1002/pro6.1089

[58] Qu Y, Zhang S, Qu Y, Guo H, Wang S, Wang X, et al. Novel Gene Signature Reveals Prognostic Model in Acute Myeloid Leukemia. Front Genet. 2020;11:566024. doi: 10.3389/fgene.2020.566024 33193652 PMC7655922

[59] Schnerch D, Yalcintepe J, Schmidts A, Becker H, Follo M, Engelhardt M, et al. Cell cycle control in acute myeloid leukemia. Am J Cancer Res. 2012;2(5):508–28. 22957304 PMC3433102

[60] Bachmann M, Hennemann H, Xing PX, Hoffmann I, Möröy T. The oncogenic serine/threonine kinase Pim-1 phosphorylates and inhibits the activity of Cdc25C-associated kinase 1 (C-TAK1): a novel role for Pim-1 at the G2/M cell cycle checkpoint. J Biol Chem. 2004;279(46):48319–28. doi: 10.1074/jbc.M404440200 15319445

[61] Karimdadi Sariani O, Eghbalpour S, Kazemi E, Rafiei Buzhani K, Zaker F. Pathogenic and therapeutic roles of cytokines in acute myeloid leukemia. Cytokine. 2021;142:155508. doi: 10.1016/j.cyto.2021.155508 33810945

[62] Ferrone S, Whiteside TL. Tumor microenvironment and immune escape. Surg Oncol Clin N Am. 2007;16(4):755–74, viii. doi: 10.1016/j.soc.2007.08.004 18022543

[63] Lee J, Cho S, Hong S-E, Kang D, Choi H, Lee J-M, et al. Integrative Analysis of Gene Expression Data by RNA Sequencing for Differential Diagnosis of Acute Leukemia: Potential Application of Machine Learning. Front Oncol. 2021;11:717616. doi: 10.3389/fonc.2021.717616 34497767 PMC8419339

[64] Li X, Xu L. Exploring prognostic markers for patients with acute myeloid leukemia based on cuproptosis related genes. Transl Cancer Res. 2023;12(8):2008–22. doi: 10.21037/tcr-23-85 37701119 PMC10493802

[65] Geest CR, Coffer PJ. MAPK signaling pathways in the regulation of hematopoiesis. J Leukoc Biol. 2009;86(2):237–50. doi: 10.1189/jlb.0209097 19498045

[66] Lee S, Rauch J, Kolch W. Targeting MAPK Signaling in Cancer: Mechanisms of Drug Resistance and Sensitivity. Int J Mol Sci. 2020;21(3):1102. doi: 10.3390/ijms21031102 32046099 PMC7037308

[67] Cornel AM, Mimpen IL, Nierkens S. MHC Class I Downregulation in Cancer: Underlying Mechanisms and Potential Targets for Cancer Immunotherapy. Cancers (Basel). 2020;12(7):1760. doi: 10.3390/cancers12071760 32630675 PMC7409324

[68] Taylor BC, Balko JM. Mechanisms of MHC-I Downregulation and Role in Immunotherapy Response. Front Immunol. 2022;13:844866. doi: 10.3389/fimmu.2022.844866 35296095 PMC8920040

[69] Meshinchi S, Appelbaum FR. Structural and functional alterations of FLT3 in acute myeloid leukemia. Clin Cancer Res. 2009;15(13):4263–9. doi: 10.1158/1078-0432.CCR-08-1123 19549778 PMC2716016

[70] Nagano T, Fraser P. No-nonsense functions for long noncoding RNAs. Cell. 2011;145(2):178–81. doi: 10.1016/j.cell.2011.03.014 21496640

[71] Volpe G, Cauchy P, Walton DS, Ward C, Blakemore D, Bayley R, et al. Dependence on Myb expression is attenuated in myeloid leukaemia with N-terminal CEBPA mutations. Life Sci Alliance. 2019;2(2):e201800207. doi: 10.26508/lsa.201800207 30877232 PMC6421631

[72] Vicente C, Conchillo A, Pauwels D, Vazquez I, Garcia-Orti L, Calasanz MJ, et al. MYB overexpression is directly involved in acute myeloid leukemia pathogenesis and could constitute a new therapeutic target for patients with aberrant expression of this gene. Blood. 2009;114(22):2402–2402. doi: 10.1182/blood.v114.22.2402.2402

[73] Norvil AB, AlAbdi L, Liu B, Tu YH, Forstoffer NE, Michie AR, et al. The acute myeloid leukemia variant DNMT3A Arg882His is a DNMT3B-like enzyme. Nucleic Acids Res. 2020;48(7):3761–75. doi: 10.1093/nar/gkaa139 32123902 PMC7144950

[74] Ommen HB. Monitoring minimal residual disease in acute myeloid leukaemia: a review of the current evolving strategies. Ther Adv Hematol. 2016;7(1):3–16. doi: 10.1177/2040620715614529 26834951 PMC4713887

[75] Cluzeau T, Lemoli RM, McCloskey J, Cooper T. Measurable residual disease in high-risk acute myeloid leukemia. Cancers (Basel). 2022;14(5):1278. doi: 10.3390/cancers14051278 35267586 PMC8909238

[76] Hourigan CS, Karp JE. Minimal residual disease in acute myeloid leukaemia. Nat Rev Clin Oncol. 2013;10(8):460–71. doi: 10.1038/nrclinonc.2013.100 23799371 PMC4163748

[77] Berti M, Vindigni A. Replication stress: getting back on track. Nat Struct Mol Biol. 2016;23(2):103–9. doi: 10.1038/nsmb.3163 26840898 PMC5125612

[78] Montagnoli A, Moll J, Colotta F. Targeting cell division cycle 7 kinase: a new approach for cancer therapy. Clin Cancer Res. 2010;16(18):4503–8. doi: 10.1158/1078-0432.CCR-10-0185 20647475

[79] Vonk CM, Al Hinai ASA, Hanekamp D, Valk PJM. Molecular minimal residual disease detection in acute myeloid leukemia. Cancers (Basel). 2021;13(21):5431. doi: 10.3390/cancers13215431 34771594 PMC8582498

[80] Sharifi MJ, Xu L, Nasiri N, Ashja-Arvan M, Soleimanzadeh H, Ganjalikhani-Hakemi M. Immune-dysregulation harnessing in myeloid neoplasms. Cancer Med. 2024;13(17):e70152. doi: 10.1002/cam4.70152 39254117 PMC11386321

[81] Guarnera L, Bravo-Perez C, Visconte V. Immunotherapy in Acute Myeloid Leukemia: A Literature Review of Emerging Strategies. Bioengineering (Basel). 2023;10(10):1228. doi: 10.3390/bioengineering10101228 37892958 PMC10604866

[82] Sudholz H, Delconte RB, Huntington ND. Interleukin-15 cytokine checkpoints in natural killer cell anti-tumor immunity. Curr Opin Immunol. 2023;84:102364. doi: 10.1016/j.coi.2023.102364 37451129

[83] Fathi AT, Abdel-Wahab O. Mutations in epigenetic modifiers in myeloid malignancies and the prospect of novel epigenetic-targeted therapy. Adv Hematol. 2012;2012:469592. doi: 10.1155/2012/469592 21811504 PMC3145345

[84] Steelman LS, Pohnert SC, Shelton JG, Franklin RA, Bertrand FE, McCubrey JA. JAK/STAT, Raf/MEK/ERK, PI3K/Akt and BCR-ABL in cell cycle progression and leukemogenesis. Leukemia. 2004;18(2):189–218. doi: 10.1038/sj.leu.2403241 14737178

[85] Zhang L, Yu X, Zheng L, Zhang Y, Li Y, Fang Q, et al. Lineage tracking reveals dynamic relationships of T cells in colorectal cancer. Nature. 2018;564(7735):268–72. doi: 10.1038/s41586-018-0694-x 30479382

[86] Kantarjian H, Kadia T, DiNardo C, Daver N, Borthakur G, Jabbour E, et al. Acute myeloid leukemia: current progress and future directions. Blood Cancer J. 2021;11(2):41. doi: 10.1038/s41408-021-00425-3 33619261 PMC7900255

[87] Gómez-Llobell M, Peleteiro Raíndo A, Climent Medina J, Gómez Centurión I, Mosquera Orgueira A. Immune Checkpoint Inhibitors in Acute Myeloid Leukemia: A Meta-Analysis. Front Oncol. 2022;12:882531. doi: 10.3389/fonc.2022.882531 35530329 PMC9069679

[88] Stubbins RJ, Francis A, Kuchenbauer F, Sanford D. Management of Acute Myeloid Leukemia: A Review for General Practitioners in Oncology. Curr Oncol. 2022;29(9):6245–59. doi: 10.3390/curroncol29090491 36135060 PMC9498246

[89] Daver N, Boddu P, Garcia-Manero G, Yadav SS, Sharma P, Allison J, et al. Hypomethylating agents in combination with immune checkpoint inhibitors in acute myeloid leukemia and myelodysplastic syndromes. Leukemia. 2018;32(5):1094–105. doi: 10.1038/s41375-018-0070-8 29487386 PMC6916728

