# Supplementary material for: Gene expression profiling and pathway analysis in acute myeloid leukaemia-normal karyotype patients
Source: PLoS One. 2025 Sep 5;20(9):e0328911. doi: 10.1371/journal.pone.0328911 (PMC12412999; doi:10.1371/journal.pone.0328911)
Supplement: S1 File — (DOCX) [file pone.0328911.s001.docx]

### SI Sample size calculation

The sample size was determined as per the recommendation by Weaver (2017) based on the following calculation:

For significance level, α = 0.05 with desired power = 90% and an expected hazard ratio, HR = 0.4 (60% reduction in hazards between groups):

The expected number of events = $\frac{(z \alpha/2+ z\beta)2}{\pi1 \pi2 (log HR)2}$

= (1.96+0.842)^2^

1 x 1 (ln 0.4)^2^

2 2

= 7.851204

0.21

= 37 events (death for overall survival)


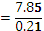


For a 5-year mortality rate of more than 77% in AML (Bethesda, 2015. The Surveillance, Epidemiology, and End Results Cancer Statistics Facts) [1] :

the expected number of patients = 37 / 0.77= 48 patients

With an estimation of 10% of potential dropouts:

The estimated sample size = 48 x 10% = **53 patients**

Hence, the sample size, based on the calculation, is 53 patients. During recruitment, 51 patients fulfilled the recruitment criteria and were included in this study.
