# Supplementary material for: Gene expression profiling and pathway analysis in acute myeloid leukaemia-normal karyotype patients
Source: PLoS One. 2025 Sep 5;20(9):e0328911. doi: 10.1371/journal.pone.0328911 (PMC12412999; doi:10.1371/journal.pone.0328911)
Supplement: S2 File — (DOCX) [file pone.0328911.s002.docx]

### SII Subject inclusion and exclusion criteria

The AML-NK patient inclusion criteria were:

i. AML-NK patients at presentation as confirmed by the conventional cytogenetics based on at least 20 bone marrow metaphases (Döhner, Andrew H Wei, et al., 2022).

ii. AML-NK patients> 12 years of age.

The patient exclusion criteria were:

i. AML patients who present with abnormal karyotype.

ii. AML patients with inconclusive karyotype results or with no metaphase.

The control inclusion criteria were:

i. Healthy adult subjects with full blood count parameters within normal limits.

ii. No history of cancer or blood disorders.

No immediate family history of cancer or blood disorders.
