# Supplementary material for: Gene expression profiling and pathway analysis in acute myeloid leukaemia-normal karyotype patients
Source: PLoS One. 2025 Sep 5;20(9):e0328911. doi: 10.1371/journal.pone.0328911 (PMC12412999; doi:10.1371/journal.pone.0328911)
Supplement: S3 File — (DOCX) [file pone.0328911.s003.docx]

### SIII DEG Pipeline

**DESeq2 Pipeline**

*Note: pipeline customisation are highlighted in yellow*

library(readr)

library(tximport)

library(DESeq2)

library(ggplot2)

library(GenomicFeatures)

library(data.table)

library(apeglm)

library(ggplot2)

library(EnhancedVolcano)

library(EnhancedVolcano)

txdb = makeTxDbFromGFF(gencodeGTF, format = "gtf", dataSource = "Gencode_v28" , organism = "Homo sapiens")

k <- keys(txdb, keytype="TXNAME")

gencodeGTF = c("/media/neo/Data/db/refanno/gencode.v28.annotation.gtf")

txdb = makeTxDbFromGFF(gencodeGTF, format = "gtf", dataSource = "Gencode_v28" , organism = "Homo sapiens")

k <- keys(txdb, keytype="TXNAME")

tx2gene <- select(txdb, k, "GENEID", "TXNAME")

head(tx2gene)

countdata <- read.table("MajorFusion_NoFusion_counts.txt", header=TRUE, row.names = 1)

View(countdata)

countdata <- countdata[ ,6:ncol(countdata)]

colnames(countdata) <- c("P2","P4","P8","P12","P13","P15","P20","P22","P23","P28","P30","P43","P45","P47","P51","P1","P3","P5","P6","P7","P9","P11","P14","P21","P24","P25","P34","P40","P44","P46","P49","P50")

countdata <-as.matrix(countdata)

condition <- factor(c(rep("Major Fusion",15), rep("No Fusion",17)))

coldata <- data.frame(row.names = colnames(countdata), condition)

coldata$condition <- relevel(coldata$condition,"No Fusion")

dds <- DESeqDataSetFromMatrix(countData = countdata, colData = coldata, design = ~condition)

dds

dds <- dds[ rowSums(counts(dds)) >= 10, ]

dds

dds = DESeq(dds)

cbind(resultsNames(dds))

res <- results(dds, name = "condition_Major.Fusion_vs_No.Fusion", alpha = 0.01)

summary(res)

resLFC = lfcShrink(dds, coef = "condition_Major.Fusion_vs_No.Fusion", type="apeglm")

png("Major Fusion vs No Fusion - MA-plot.png", width=7, height=5, units = "in", res = 300)

plotMA(resLFC, alpha = 0.01, ylim=c(-6,6),

main = "MA-plot for the shrunken log2 fold changes")

dev.off()

rld = rlog(dds)

vsd = vst(dds)

pcaData = plotPCA(rld, intgroup=c("condition"), returnData=TRUE)

percentVar = round(100 * attr(pcaData, "percentVar"))

png("Major Fusion vs No Fusion PCA Plot-rlog.png", width=10, height=10, units = "in", res = 300)

ggplot(pcaData, aes(PC1, PC2, colour = condition)) +

geom_point(size = 3) + theme_bw() +

scale_color_manual(values = c("blue", "red")) +

geom_text_repel(aes(label = name), nudge_x = -1, nudge_y = 0.2, size = 3) +

ggtitle("Principal Component Analysis (PCA) Major Fusion vs No Fusion", subtitle = "rlog transformation") +

xlab(paste0("PC1: ",percentVar[1],"% variance")) +

ylab(paste0("PC2: ",percentVar[2],"% variance"))

dev.off()

pcaData = plotPCA(vsd, intgroup=c("condition"), returnData=TRUE)

View(pcaData)

percentVar = round(100 * attr(pcaData, "percentVar"))

png("Major Fusion vs No Fusion PCA Plot-vst.png", width=10, height=10, units = "in", res = 300)

ggplot(pcaData, aes(PC1, PC2, colour = condition)) +

geom_point(size = 3) + theme_bw() +

scale_color_manual(values = c("blue", "red")) +

geom_text_repel(aes(label = name), nudge_x = -1, nudge_y = 0.2, size = 3) +

ggtitle("Principal Component Analysis (PCA) Major Fusion vs No Fusion", subtitle = "vst transformation") +

xlab(paste0("PC1: ",percentVar[1],"% variance")) +

ylab(paste0("PC2: ",percentVar[2],"% variance"))

dev.off()

pCutoff = 0.01

FCcutoff = 1.0

p = EnhancedVolcano(data.frame(res), lab = NA, x = 'log2FoldChange', y = 'padj',

xlab = bquote(~Log[2]~ 'fold change'), ylab = bquote(~-Log[10]~adjusted~italic(P)),

pCutoff = pCutoff, FCcutoff = FCcutoff, pointSize = 1.0, labSize = 2.0,

title = "Volcano plot", subtitle = "Major Fusion vs. No Fusion",

caption = paste0('log2 FC cutoff: ', FCcutoff, '; p-value cutoff: ', pCutoff, '\nTotal = ', nrow(res), ' variables'),

legend=c('NS','Log2 FC','Adjusted p-value', 'Adjusted p-value & Log2 FC'),

legendPosition = 'bottom', legendLabSize = 14, legendIconSize = 5.0)

png("Major Fusion vs No Fusion - VolcanoPlots.png", width=7, height=7, units = "in", res = 300)

print(p)

dev.off()

normCounts = as.data.frame(counts(dds, normalized = TRUE))

write.csv(normCounts, file = "Norm_Counts.csv")

library("pheatmap")

library("RColorBrewer")

rld_mat <- assay(rld)

write.csv(rld_mat, file="rlog_Counts.csv")

rld_cor <- cor(rld_mat)

write.csv(rld_cor, file="rlog_Correlation.csv")

hmcol <- colorRampPalette(c("red","yellow","darkgreen"))(30)

png("Major Fusion vs No Fusion Correlation_Heatmap.png", width=10, height=10, units = "in", res = 300)

pheatmap(rld_cor,main = "KMT2A vs No Fusion Correlation Analysis",cluster_rows=F, cluster_cols=F, fontsize = 12, color = hmcol)

dev.off()

res <- res[order(res$padj), ]

write.csv(res, file="All-DEGs-Raw.csv")

resSig <- subset(res, padj<0.01 & abs(log2FoldChange)>1 & baseMean > 100)

resSig <- resSig[order(resSig$padj), ]

View(resSig)

write.csv(resSig, file = "All_Significant_Genes_List.csv")

norm_counts = counts(dds, normalized = TRUE)

hmcol2 <- colorRampPalette(c("green","red"))(30)

png("Major Fusion vs No Fusion Hierarchical_Clustering.png", width=12, height=15, units = "in", res = 300)

forHeatmap <- resSig[1:100,]

write.csv(forHeatmap, file = "Top_100_Significant_Gene_List.csv")

norm_0Esig <- norm_counts[rownames(forHeatmap),]

View(norm_0Esig)

mat_col <- data.frame(sample_condition = colData(rld)[,c("condition")])

pheatmap(norm_0Esig, color=hmcol2, show_rownames=T, labels_row = forHeatmap$SYMBOL,fontsize = 12, scale = "row", main = "Top 100 Significant Genes")

dev.off()

rld_mat <- assay(rld)

rld_cor <- cor(rld_mat)

hmcol <- colorRampPalette(c("red","yellow","darkgreen"))(30)

png("Major Fusion vs No Fusion Correlation_Heatmap.png", width=10, height=10, units = "in", res = 300)

pheatmap(rld_cor,main = "Major Fusion vs No Fusion Correlation Analysis",cluster_rows=F, cluster_cols=F, fontsize = 12, color = hmcol)

dev.off()

savehistory("/media/neo/Data/Angeli/DEG_RScript.txt")
