# Supplementary material for: Gene expression profiling and pathway analysis in acute myeloid leukaemia-normal karyotype patients
Source: PLoS One. 2025 Sep 5;20(9):e0328911. doi: 10.1371/journal.pone.0328911 (PMC12412999; doi:10.1371/journal.pone.0328911)
Supplement: S4 File — (DOCX) [file pone.0328911.s004.docx]

### SIV The RT-qPCR protocols

**Differential gene expression (deg) validation by real-time quantitative RT-QPCR (RT-QPCR)**

Primer design for DEG

Primers were synthesised by 1st BASE Next-Generation Sequencing Services by Apical Scientific Sdn Bhd (Selangor, Malaysia). The steps for primer design are as follows:

The Gene RefSeq Information was searched, and the longest isoform was chosen at the following link: <https://www.ncbi.nlm.nih.gov/gene>. The gene sequence was derived using the Get the IDT Primer Quest at the following link:<https://sg.idtdna.com/pages/tools/primerquest?utm_source=google&utm_medium=cpc&utm_campaign=ga_primerquest&utm_content=ad_group_primerquest&gclid=CjwKCAiAvriMBhAuEiwA8Cs5ldJ6wTdiVTkBrqA6vM-UskJ8UlKb2iEpObBEhT7qqJOUWNy4Scdf9RoChN8QAvD_BwE>.

The sequence was downloaded using the Gene RefSeq, and the design (qPCR two primers Intercalating dyes) was chosen. A primer specificity check was done using Primer Blast using the following link: <https://www.ncbi.nlm.nih.gov/tools/primer-blast/>. Primer Dimer Check was done using OligoEvaluator using the following link: <http://www.oligoevaluator.com/OligoCalcServlet>. The list of primers used in the DEG and the RT-qPCR are listed in Table SIV.1.

Table SIV.1: List of primers used in the DEG RT-qPCR

| **Primer name** | **Primer sequence (5'–3')** | **Type** |
| --- | --- | --- |
| *B2M* - F | CAG CGT ACT CCA AAG ATT CAG G | Reference gene |
| *B2M* - R | AGT CAA CTT CAA TGT CGG ATG G | Reference gene |
| *GAPDH*-F | GGA GCG AGA TCC CTC CAA AAT | Reference gene |
| *GAPDH-*R | GGC TGT TGT CAT ACT TCT CAT GG | Reference gene |
| *TDP2-*F | TGC TGC GGA ACG AAT GAA | DEG |
| *TDP2-*R | GGT AAC CTC TCG ATC CCT TAG A | DEG |
| *STK40-*F | AGG GAG ACT GTG GTA ATC TTC T | DEG |
| *STK40-*R | GGT TAT CCG ATG TGT CCT CTT G | DEG |
| *PACS1-*F | GAA GAC CCG GAG GAA ACT AAC | DEG |
| *PACS1-*R | AAG CCC ACC TCA TCT GAA AC | DEG |
| *OAZ2-*F | CTG CTA GAG TTT GCT GAA GAG A | DEG |
| *OAZ2-*R | GCC TGG ACG TAC AAT CTC AA | DEG |
| *TCF20-*F | GGG AGC AAA GGA AAC AAG AAT G | DEG |
| *TCF20-*R | GGC TCA GTT CTG CTC GTA AA | DEG |
| *CRISPLD1-*F | CTG CTG GCT GTT TGG ATA GT | DEG |
| *CRISPLD1-*R | CCA GCC ACC ATC ATT GTC TAT TA | DEG |

**Real-time quantitative RT-PCR (qRT-PCR)**

To optimise the qPCR primer, a range of annealing temperatures around the calculated primer melting temperature (Tm) were tested. A standard curve using serial dilutions of template cDNA was performed to verify the efficiency of the primers. The specificity of the primers was evaluated using a melt curve. The reactions were prepared using the Maxima SYBR Green qPCR Master Mix (ThermoFisher Scientific, Waltham, MA, USA) according to the manufacturer's instructions using the cDNA. The cDNA synthesis was performed using Maxima First Strand cDNA Synthesis Kit (ThermoFisher Scientific, Waltham, MA, USA). Gene expression levels were quantified relative to the reference genes GAPDH and B2M.

**cDNA synthesis**

Samples were diluted to 25ng/µL, and in a reaction tube, the reverse transcription (RT) reagents, according to Table SIV.2, were added to the reaction tube. The reactions were mixed thoroughly by gentle tapping, spinning down, and incubating for 2 minutes at 37 ^0^C in the thermal cycler. Next, the reactions were placed on ice for 3 minutes.

Table SIV.2: Reverse transcription reagent preparation.

| **Reagent** | **1x** |
| --- | --- |
| 10X reaction buffer with MgCl_2_ | 1 |
| Dnase I | 1 |
| RNA (200 ng) | 8 |
| **Total** | **10** |

Next, the reagents, according to Table SIV.3, were prepared and added to the reaction tubes, incubated at 25 0C for 10 minutes, followed by 50 0C for 15 minutes, and finally at 85 ^0^C for 5 minutes. Then, 20µl nuclease-free H2O was added to the reaction and placed on ice.

Table SIV.3: Enzyme mix preparation.

| **Reagent** | **1x** |
| --- | --- |
| 5X reaction mix | 4 |
| Maxima enzyme mix | 2 |
| H_2_O | 4 |

**Optimization step (melting curve & standard curve & primer efficiency)**

Standard curves using serially diluted cDNA (0.04ng – 10ng) and no template control (NTC) were used to check if there was any contamination, and RT negative control was used to check for genomic DNA contamination. The primer was diluted to 5 µM (25ul of 10uM stock + 25ul nuclease-free H2O). Serial dilutions (5x) using 2 times diluted cDNA (control sample) were performed as follows: i) first point of dilution: stock cDNA, ii) second point of dilution: 2 µL first point diluted cDNA + 8 µL H2O, iii) third point of dilution: 2 µL second point diluted cDNA + 8 µL H2O, iv) fourth point of dilution: 2 µL third point diluted cDNA + 8 µL H2O, and v) fifth point of dilution: 2 µL fourth point diluted cDNA + 8 µL H2O. The qPCR master mix was prepared according to Table SIV.4.

Table SIV.4: qPCR master mix.

| **Reagent** | **Volume (µl)** |
| --- | --- |
| Nuclease free water | 7.5 |
| Maxima SYBR Green qPCR Master Mix (2x) | 12.5 |
| Forward Primer, 5 µM (final con. 300nM) | 1.5 |
| Reverse Primer, 5 µM (final con. 300nM) | 1.5 |
| cDNA (50ng – 0.08ng) | 2.0 |
| **Total** | **25.0** |

Then, 18 µL master mix was aliquoted into strip tubes, and 2 µL of cDNA was put into the respective tubes, as shown in Table SIV.5.

Table SIV.5: Layout for mastermix aliquot.

| Gene A | 1 | 1 | 1 | 2 | 2 | 2 | 3 | 3 |
| --- | --- | --- | --- | --- | --- | --- | --- | --- |
|  | 3 | 4 | 4 | 4 | 5 | 5 | 5 | NTC |
|  | NTC | RT | RT |  |  |  |  |  |
| Gene B | 1 | 1 | 1 | 2 | 2 | 2 | 3 | 3 |
|  | 3 | 4 | 4 | 4 | 5 | 5 | 5 | NTC |
|  | NTC | RT | RT |  |  |  |  |  |

Next, the thermal cycler was set according to Table SIV.6 for melting curve analysis.

Table SIV.6: PCR cycle

| **Steps** | **Temperature** | **Duration** | **Cycle** |
| --- | --- | --- | --- |
| Initial denaturation | 95 | 10 min | 1X |
| Amplification | 95 | 15 sec | 40X |
|  | 60 | 1 min |  |
| Melt Curve Analysis | 95 | 30 sec | 1X |
|  | 65 | 30 sec |  |
|  | 95 | 30 sec |  |

Data analysis includes melting curves, standard curves, and primer efficiency reviews. For the gene expression protocol, a calibrator (4 healthy controls), unknown (6 AML-NK patients with dysregulated gene as listed in Table 3.18 (Chapter 3) and NTC were used. Primers were diluted to 5 µM (10µl of 10µM stock + 10ul nuclease-free H2O), and qPCR master mix was prepared according to Table C.7. Then 18 µL master mix was aliquoted into strip tubes, and 2 µL of cDNA into the respective tubes as depicted in Table X. Next, the thermal cycler was set according to Table X in which fluorescent data was collected during the amplification step.

Table SIV.7: qPCR master mix

| **Reagent** | **Gene expression (µl)** | |
| --- | --- | --- |
|  | **1X** | **9X** |
| Nuclease free water | 4 | 36 |
| 2X brilliant III ultra-fast SYBR green qPCR master mix | 10 | 90 |
| Forward primer, 5µM (final concentration of 500 nM) | 2 | 18 |
| Reverse primer, 5µM (final concentration of 500 nM) | 2 | 18 |
| cDNA (10ng) | 2 |  |
| **Total** | **20** | **18** |

Table SIV.8: Mastermix aliquot

| Reference gene 1 | C1 | C1 | C1 | U1 | U1 | U1 | NTC | NTC |
| --- | --- | --- | --- | --- | --- | --- | --- | --- |
| Reference gene 2 | C1 | C1 | C1 | U1 | U1 | U1 | NTC | NTC |
| Gene of interest 1 | C1 | C1 | C1 | U1 | U1 | U1 | NTC | NTC |
| Gene of interest 2 | C1 | C1 | C1 | U1 | U1 | U1 | NTC | NTC |
| Gene of interest 3 | C1 | C1 | C1 | U1 | U1 | U1 | NTC | NTC |

C- Calibrator, U- Unknown

Table SIV.9: PCR cycles.

| **Steps** | **Temperature (**$\boldsymbol{℃}$**)** | **Duration** | **Cycle** |
| --- | --- | --- | --- |
| Initial denaturation | 95 | 10 min | 1X |
| Amplification | 95 | 15 sec | 40X |
|  | 60 | 1 min |  |
| Melt Curve Analysis | 95 | 30 sec | 1X |
|  | 65 | 30 sec |  |
|  | 95 | 30 sec |  |

**Data analysis using the 2^-ΔΔCT^ method**

The 2−ΔΔCT method was used to determine the amount of each gene present relative to each sample. The ΔCt was calculated by subtracting the reference gene's Ct value from the target gene's Ct value. The ΔΔCt was calculated by subtracting the ΔCt of the control sample from the ΔCt of each treated sample. Compared to the control, the fold change of the treated target gene is represented by the negative value of this subtraction (CT), which is utilised as the exponent 2 in the equation. The equations are as follows: Normalising to reference gene (ΔCt = Ct gene of interest – Ct reference gene) and calculate fold change 2-ΔΔCt (ΔΔCt = ΔCt unknown - ΔCt calibrator) as depicted in Figures C.1 and C.2. To obtain statistical significance, the minimum number of samples per group was three (Ref for all ΔΔCt).


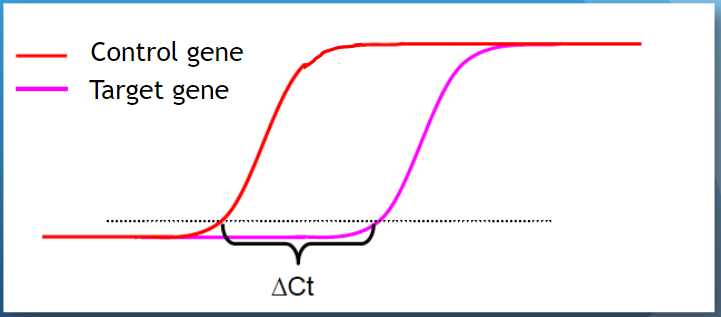


Figure C SIV.1. The ΔϹt is depicted in the diagram that represents the control and target gene.


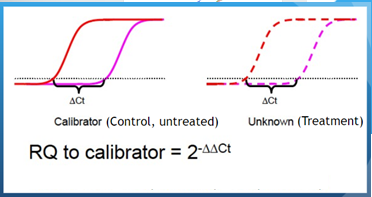


Figure SIV.2. The relative quantification (RQ) = 2-ΔΔϹt is depicted in the diagram that represents the calibrator (healthy control) and unknown (AML-NK).

**Sanger Sequencing primer design and sample preparation**

**Primer design for breakpoint validation**

Primers were synthesised by 1st BASE and designed using the online PrimerQuest Tool on the IDT website (<https://sg.idtdna.com/Primerquest/Home/Index>).

**Breakpoint validation**

The sequence was searched in NCBI by typing the gene name or NCBI ID. Then, genomic regions, transcripts, and product tabs were selected, and the genomic sequence was reviewed for reference selection. Then, the FASTA file was selected. The primer was designed using the IDT Primer Quest (<https://sg.idtdna.com/Primerquest/Home/Index>), and Primer Specificity Check was done using the Primer Blast (<https://www.ncbi.nlm.nih.gov/tools/primer-blast/>).

The primer dimer check was done using Multiple Primer Analyzer (<https://www.thermofisher.com/my/en/home/brands/thermo-scientific/molecular-biology/molecular-biology-learning-center/molecular-biology-resource-library/thermo-scientific-web-tools/multiple-primer-analyzer.html>).

**Amplification and analysis of DNA fragments**

The polymerase chain reaction (PCR) amplified specific nucleic acid sequences. The purified PCR reactions were sent to Apical Scientific Sdn Bhd (Selangor, Malaysia) for Sanger sequencing.

**cDNA synthesis**

The cDNA synthesis was done using AffinityScript QPCR cDNA Synthesis Kit (Agilent Technologies, Santa Clara, CA, USA). The first-strand cDNA synthesis reaction was prepared in a microcentrifuge tube by adding the components listed in Table C.10 in order.

Table SIV.10. First-strand cDNA synthesis reagents

| **Reagent** | **1X** |
| --- | --- |
| RNAse-free H20+ sample | 6 |
| First-strand master mix (2x) | 10 |
| Random primers (0.1 µg/µl) | 3 |
| AffinityScript RT/Rnase block enzyme mixture | 1 |
| **Total** | **20** |

The reaction at 25°C was incubated for 5 minutes to allow primer annealing, followed by 15 minutes at 42°C to enable cDNA synthesis. Next, the reactions were incubated at 95°C for 5 minutes to terminate the cDNA synthesis reaction. The completed first-strand cDNA synthesis reaction was placed on ice for immediate use in qPCR. The cDNA was diluted to 20ng/μl.

**PCR Reaction Preparation**

The PCR reactions were prepared using the Phusion High-Fidelity PCR Master Mix (Thermo Scientific) according to the manufacturer's instructions using 80ng of DNA and 0.5 µM forward and reverse primer. PCR was performed with the following cycling conditions: an initial denaturation at 98 °C for 30 s, 30 denaturation-annealing-elongation cycles at 98 °C for 10 s, primer-specific annealing temperature for 30 s and 72 °C for 15 s, followed by a final elongation step at 72 °C for 10 min. Then, the PCR products were separated according to their size by TapeStation Systems (Agilent Technologies, Santa Clara, CA, USA).

**Sample preparation of Sanger Sequencing**

The PCR products amplified as described above were purified to remove PCR components, like primers and unincorporated dNTPs. For this purpose, PCR reactions were purified using Expin™ PCR SV mini (GeneAll, Seoul, Korea) according to the manufacturer's protocol. Finally, the dissolved DNA and an aliquot of 10 µM forward or reverse primer were sent to 1st BASE Next-Generation Sequencing Services by Apical Scientific Sdn Bhd (Selangor, Malaysia). The software Chromas 2.6.6 (Technelysium Pty Ltd, Brisbane, Australia) was used to visualise the chromatographs.
