# Supplementary material for: Gene expression profiling and pathway analysis in acute myeloid leukaemia-normal karyotype patients
Source: PLoS One. 2025 Sep 5;20(9):e0328911. doi: 10.1371/journal.pone.0328911 (PMC12412999; doi:10.1371/journal.pone.0328911)
Supplement: S5 File — (DOCX) [file pone.0328911.s005.docx]

### SV Patient demographic and clinical characteristics.

Table SV.1. Patient demographic and clinical characteristics

| **Characteristics** | **Male** | **Female** | **Total** | **P-value** |
| --- | --- | --- | --- | --- |
|  | **n (%)** | **n (%)** | **n (%)** |  |
| Total | 27 (54) | 24 (47) | 51 (100) |  |
| **Age (years)** |  |  |  |  |
| Median | 47 | 49 | 49 | 0.546 ‡ |
| Range (IQR) | 22-57 | 32-57 | 27-57 |  |
| **Age groups (years)** |  |  |  |  |
| Below 60 | 21 (41) | 20 (39) | 41 (80) | 0.731 † |
| Above 60 | 6 (12) | 4 (8) | 10 (20) |  |
| **Ethnicity** |  |  |  |  |
| Malay | 16 (31) | 16 (31) | 32 (62) | 0.845 † |
| Chinese | 8 (16) | 5 (10) | 13 (26) |  |
| Indian | 3 (6) | 3 (6) | 6 (12) |  |
| **TWBC (10^9^/L)** |  |  |  |  |
| Median | 58.30 | 29.00 | 39.50 | 0.763 ‡ |
| Range (IQR) | 8.7-80 | 14-111 | 13-100 |  |
| **Haemoglobin (****g/dL)** |  |  |  |  |
| Mean | 8.4 | 8.2 | 8.278 | 0.887 ‡ |
| SD | 2.4 | 2.3 | 2.3 |  |
| **Platelet (10^9^/L)** |  |  |  |  |
| Median | 52 | 71 | 63 | 0.089 ‡ |
| Range (IQR) | 27-81 | 38-87 | 31-86 |  |
| **Genotype (*FLT3* and *NPM1*)** |  |  |  |  |
| *FLT3-*ITD*/NPM1^mut^* | 3 (7) | 6 (13) | 9 (20) | 0.043 †* |
| *FLT3-*ITD*/NPM1^wt^* | 6 (13) | 0 (0) | 6 (13) |  |
| *FLT3^wt^/NPM1^mut^* | 4 (9) | 5 (11) | 9 (20) |  |
| *FLT3^wt^/NPM1^wt^* | 13 (28) | 9 (20) | 22 (48) |  |
| Genotype not available | 1 (2) | 4 (8) | 5 (10) |  |
| **ELN 2017** |  |  |  |  |
| Good prognosis | 4 (8) | 5 (10) | 9 (18) | 0.050†♦ |
| Intermediate prognosis | 16 (31) | 15 (29) | 31 (61) |  |
| Adverse prognosis | 6 (12) | 0 (0) | 6 (12) |  |
| Not available | 1 (2) | 4 (8) | 5 (10) |  |
| **Progenitor marker** |  |  |  |  |
| CD34^pos^ | 23 (45) | 11 (22) | 34 (67) |  |
| **Aberrant antigen** |  |  |  |  |
| CD56^pos^ | 3 (6) | 3 (6) | 6 (12) | 0.737 †♣ |
| CD19^pos^ | 3 (6) | 1 (2) | 4 (8) |  |
| CD7^pos^ | 6 (12) | 8 (16) | 14 (27) |  |
| CD7+56^pos^ | 1 (2) | 2 (4) | 3 (6) |  |
| No aberrant antigen | 14 (27) | 10 (20) | 24 (47) |  |
| **Clinical Outcome** |  |  |  |  |
| CR1 | 11 (22) | 15 (29) | 26 (51) |  |
| Refractory | 12 (24) | 7 (14) | 19 (37) |  |
| ED | 4 (8) | 2 (4) | 6 (12) |  |
| **Survival status** |  |  |  |  |
| Alive | 7 (14) | 11 (22) | 18 (35) |  |
| Dead | 20 (39) | 13 (29) | 33 (65) |  |
| **OS (Months)** |  |  |  |  |
| Median | 3.92 | 10.27 | 6.23 |  |
| Range (IQR) | 1.94-10.51 | 4.73-22.20 | 2.83-13.88 |  |
| OS < 5 years | 20 (39) | 13 (26) | 33 (65) |  |
| OS ≥ 5 years | 7 (14) | 11 (22) | 18 (35) |  |
| **Treatment Regimen** |  |  |  |  |
| **Induction protocol** |  |  |  |  |
| DA 3+7 | 19 (37) | 19 (37) | 38 (75) |  |
| IA 3+7 | 2 (4) | 0 | 2 (4) |  |
| MA 3+7 | 6 (12) | 5 (10) | 11 (22) |  |
| **History of SCT** |  |  |  |  |
| Allo-SCT | 5 (26) | 10 (53) | 15 (79) |  |
| Auto-SCT | 0 (0) | 1 (5) | 1 (5) |  |
| Haplo-SCT | 2 (11) | 1 (5) | 3 (16) |  |
| **Relapsed Status** [1] |  |  |  |  |
| No relapse | 20 (44) | 15 (33) | 35 (78) |  |
| Relapsed | 3 (7) | 7 (16) | 10 (22) |  |

[1] Early death patients (n=6) were not included in this data.

† Fisher's Exact Test

‡ Mann-Whitney Test

*The Fisher's exact test done to examine the association of the genotypes (*FLT3*-ITD/*NPM1^mu^*^t^, *FLT3-*ITD/ *NPM1^w^*^t^, *FLT3^wt/^NPM1^mut^* and *FLT3^wt^/NPM1^wt^*) and gender (male and female) was statistically significant (p=0.043)

♣ The Fisher's exact test done to examine the association of the aberrant antigen expression (CD56^pos^, CD19^pos^, CD7^pos^, CD7+56^pos^ and no aberrant antigen expression) and gender (male and female) was statistically insignificant (p=0.737)

♦ The Fisher's exact test done to examine the association of ELN 2017 classifications (good, intermediate, and adverse prognoses) and gender (male and female) was statistically significant (p=0.050).
