# Supplementary material for: Gene expression profiling and pathway analysis in acute myeloid leukaemia-normal karyotype patients
Source: PLoS One. 2025 Sep 5;20(9):e0328911. doi: 10.1371/journal.pone.0328911 (PMC12412999; doi:10.1371/journal.pone.0328911)
Supplement: S6 File — (DOCX) [file pone.0328911.s006.docx]

### SVI Quality control data of transcriptome sequencing

**Quality control data**

**Table SVI.1 Quantity and quality measurements using the NanoDrop ND-1000 UV-VIS Spectrophotometer**

| **ID** | **Sample Type** | **RNA** | | | | **DNA** | | |
| --- | --- | --- | --- | --- | --- | --- | --- | --- |
|  |  | **Conc (ng/ul)** | **A260/A280** | **A260/A230** | **RIN** | **Conc (ng/ul)** | **A260/A280** | **A260/A230** |
| H1 | Healthy Donor | 103.6 | 1.891 | 1.919 | 8.6 | 55 | 1.719 | 1.325 |
| H2 | Healthy Donor | 79.2 | 1.8 | 1.737 | 8.4 | 79 | 1.717 | 1.927 |
| H3 | Healthy Donor | 199.2 | 1.894 | 1.407 | 8.3 | 139 | 1.727 | 2 |
| H4 | Healthy Donor | 84 | 1.826 | 1.963 | 8.3 | 50.5 | 1.741 | 1.712 |
| H5 | Healthy Donor | 142.8 | 1.889 | 1.911 | 8.6 | 216 | 1.749 | 1.655 |
| H6 | Healthy Donor | 134.4 | 1.826 | 2.1 | 8.9 | 69.5 | 1.675 | 1.904 |
| H7 | Healthy Donor | 88.8 | 1.835 | 1.433 | 8.5 | 91.5 | 1.726 | 2.08 |
| H8 | Healthy Donor | 101.6 | 1.91 | 1.539 | 9.2 | 340 | 1.766 | 2.105 |
| H9 | Healthy Donor | 107.2 | 1.956 | 1.29 | 8.9 | 338.5 | 1.754 | 1.934 |
| H10 | Healthy Donor | 113.2 | 1.838 | 2.16 | 9.2 | 73.5 | 1.709 | 1.96 |
| H11 | Healthy Donor | 50.4 | 1.826 | 1.586 | 8 | 57 | 1.701 | 1.869 |
| H12 | Healthy Donor | 94.4 | 1.983 | 1.242 | 9.2 | 207.5 | 1.797 | 2.139 |
| P6 | Patient | 186.8 | 1.883 | 1.938 | 9.4 | 169.5 | 1.756 | 2.093 |
| P9 | Patient | 123.2 | 1.867 | 1.03 | 9.1 | 156 | 1.743 | 1.914 |
| P10 | Patient | 114.8 | 1.876 | 2.05 | 8.8 | 88.5 | 1.67 | 1.135 |
| P14 | Patient | 139.2 | 1.902 | 1.989 | 8.2 | 228 | 1.714 | 1.443 |
| P25 | Patient | 88 | 1.85 | 1.61 | 8.9 | 137.5 | 1.763 | 1.95 |
| P29 | Patient | 194.4 | 1.869 | 1.389 | 9 | 166.5 | 1.734 | 1.571 |
| P31 | Patient | 166.8 | 1.904 | 1.87 | 8.2 | 281.5 | 1.77 | 2.011 |
| P32 | Patient | 43.6 | 1.982 | 0.356 | 9.4 | 25.6 | 1.73 | 0.571 |
| P37 | Patient | 148.4 | 1.864 | 1.922 | 9.6 | 129.5 | 1.75 | 1.65 |
| P47 | Patient | 132.4 | 1.849 | 1.871 | 8.3 | 110 | 1.705 | 1.583 |
| P48 | Patient | 72 | 1.721 | 1.738 | 8.6 | 145.5 | 1.774 | 1.785 |
| P49 | Patient | 72 | 1.856 | 1.364 | 8.7 | 50.5 | 1.53 | 0.643 |

**Table SVI.2 Library preparation quality assessment with the 2100 Bioanalyzer DNA 1000 Assay and TapeStation System (Screentape)**

| **ID** | **Sample type** | **RNA** | | | | **Library Prep** | | |
| --- | --- | --- | --- | --- | --- | --- | --- | --- |
|  |  | **Stock Conc (ng/ul)** | **A260/280** | **A260/230** | **RIN** | **Starting conc. (ng/ul)** | **Index** | **Conc (ng/ul)** |
| H1 | Healthy Donor | 105.2 | 1.865 | 1.801 | 8.6 | 23.6 | E02 | 20 |
| H2 | Healthy Donor | 79 | 1.8 | 1.737 | 8.4 | 21.2 | D01 | 68.6 |
| H3 | Healthy Donor | 193.6 | 1.876 | 1.399 | 8.3 | 22.8 | F02 | 15.5 |
| H4 | Healthy Donor | 84 | 1.826 | 1.963 | 8.3 | 20.8 | B01 | 35.8 |
| H5 | Healthy Donor | 133.6 | 1.876 | 1.92 | 8.6 | 21.2 | G02 | 16.1 |
| H6 | Healthy Donor | 134 | 1.826 | 2.1 | 8.9 | 21.6 | G01 | 54.2 |
| H7 | Healthy Donor | 88 | 1.835 | 1.433 | 8.5 | 20.8 | C01 | 58.6 |
| H8 | Healthy Donor | 101 | 1.91 | 1.539 | 9.2 | 24 | G02 | 91 |
| H9 | Healthy Donor | 107 | 1.956 | 1.39 | 8.9 | 24 | F02 | 50.4 |
| H10 | Healthy Donor | 113 | 1.838 | 2.16 | 9.2 | 22.4 | F01 | 60 |
| H11 | Healthy Donor | 50 | 1.826 | 1.586 | 8 | 20.5 | E01 | 80 |
| H12 | Healthy Donor | 94 | 1.983 | 1.242 | 9.2 | 23.6 | H02 | 77 |
| P1 | Diagnostic | 609 | 1.819 | 1.984 | 7.8 | 20.4 | H02 | 43.2 |
| P10 | Diagnostic | 593.2 | 1.831 | 2.026 | 7.9 | 25.6 | F01 | 25.8 |
| P10 | CR1 | 115.6 | 1.841 | 2.05 | 8.8 | 22.4 | E01 | 16 |
| P11 | Diagnostic | 983 | 1.551 | 1.392 | 7.6 | 19.2 | A01 | 49.4 |
| P12 | Diagnostic | 984 | 1.537 | 1.596 | 8.5 | 22.8 | F01 | 51 |
| P13 | Diagnostic | 591 | 1.828 | 1.932 | 8.1 | 23.2 | B01 | 45.8 |
| P14 | Diagnostic | 796 | 1.717 | 1.876 | 8.3 | 22.4 | B01 | 34.4 |
| P14 | CR1 | 144 | 1.875 | 1.957 | 8.2 | 20.4 | A01 | 20.6 |
| P15 | Diagnostic | 584 | 1.815 | 2.035 | 7.5 | 22 | H01 | 46 |
| P16 | Diagnostic | 973 | 1.551 | 1.654 | 7.8 | 24.4 | C02 | 89 |
| P17 | Diagnostic | 973 | 1.549 | 1.525 | 7.7 | 23.2 | G01 | 47 |
| P18 | Diagnostic | 276 | 1.92 | 1.5 | 9.2 | 9.5 | D01 | 17.3 |
| P19 | Diagnostic | 344 | 1.96 | 1.6 | 9.3 | 8.8 | B01 | 18.6 |
| P2 | Diagnostic | 226 | 1.98 | 1.5 | 9.4 | 9.3 | E01 | 42 |
| P20 | Diagnostic | 1101 | 1.135 | 1.052 | 7.4 | 22.8 | A02 | 82.2 |
| P21 | Diagnostic | 34 | 1.83 | 1.323 | 8.8 | 20.8 | D02 | 47.4 |
| P22 | Diagnostic | 213 | 1.93 | 1.8 | 9.2 | 8.9 | A01 | 31.8 |
| P23 | Diagnostic | 201 | 1.881 | 1.091 | 8.5 | 25.6 | H01 | 88.8 |
| P24 | Diagnostic | 704 | 1.77 | 1.831 | 8.3 | 24 | B02 | 68.8 |
| P25 | Diagnostic | 394 | 1.852 | 1.841 | 8.2 | 24 | F01 | 25.6 |
| P25 | CR1 | 88 | 1.857 | 1.824 | 8.9 | 22.4 | E01 | 43.8 |
| P26 | Diagnostic | 935 | 1.602 | 1.707 | 7.4 | 26 | D01 | 53.4 |
| P27 | Diagnostic | 863 | 1.675 | 1.773 | 7.8 | 25.6 | D02 | 73.6 |
| P28 | Diagnostic | 824 | 1.694 | 1.797 | 7.4 | 24.4 | E02 | 65.6 |
| P29 | Diagnostic | 848.4 | 1.791 | 1.765 | 8.8 | 25.2 | B02 | 30.2 |
| P29 | CR1 | 192.8 | 1.861 | 1.47 | 9 | 22.4 | A02 | 47.8 |
| P3 | Diagnostic | 148 | 1.96 | 1.9 | 8.9 | 9.3 | F01 | 27 |
| P30 | Diagnostic | 1002 | 1.504 | 1.549 | 7.5 | 24.8 | C01 | 88 |
| P31 | Diagnostic | 926 | 1.652 | 1.509 | 6.6 | 23.6 | D01 | 17.5 |
| P31 | CR1 | 160 | 1.883 | 1.9 | 8.2 | 23.6 | C01 | 60.8 |
| P32 | Diagnostic | 186.4 | 1.887 | 1.887 | 8.8 | 22 | D02 | 22 |
| P32 | CR1 | 45.2 | 1.823 | 0.368 | 9.4 | 21.2 | C02 | 29.4 |
| P33 | Diagnostic | 184 | 1.866 | 2.049 | 8.7 | 20.8 | E02 | 44.8 |
| P34 | Diagnostic | 802 | 1.72 | 1.814 | 8.3 | 26 | A02 | 108 |
| P35 | Diagnostic | 992 | 1.515 | 1.573 | 8.2 | 19.6 | F02 | 35.8 |
| P36 | Diagnostic | 488 | 1.98 | 1.8 | 8.8 | 9 | A02 | 40.5 |
| P37 | Diagnostic | 717.2 | 1.793 | 1.938 | 7.8 | 23.6 | D01 | 22.6 |
| P37 | CR1 | 150.4 | 1.871 | 1.852 | 9.6 | 26.4 | C01 | 53 |
| P38 | Diagnostic | 897 | 1.643 | 1.748 | 7.2 | 25.6 | E01 | 81.2 |
| P39 | Diagnostic | 605 | 1.798 | 1.909 | 7.7 | 24 | B01 | 58.8 |
| P4 | Diagnostic | 447 | 1.856 | 1.929 | 8.2 | 24 | H02 | 31 |
| P40 | Diagnostic | 799 | 1.714 | 1.876 | 8.8 | 21.6 | C02 | 51 |
| P41 | Diagnostic | 462 | 1.838 | 1.511 | 7.7 | 25.6 | A01 | 86.2 |
| P42 | Diagnostic | 880 | 1.654 | 1.784 | 7.9 | 26 | G01 | 100 |
| P43 | Diagnostic | 912 | 1.62 | 1.723 | 7.9 | 21.2 | G02 | 68.4 |
| P44 | Diagnostic | 263 | 1.93 | 1.9 | 9.1 | 8.9 | B02 | 19.8 |
| P45 | Diagnostic | 96 | 1.875 | 1.356 | 8.3 | 21.6 | D01 | 39 |
| P46 | Diagnostic | 703 | 1.777 | 1.968 | 7.6 | 24.8 | F01 | 68.4 |
| P47 | Diagnostic | 287 | 1.96 | 1.7 | 9.2 | 9.1 | H01 | 26 |
| P47 | CR1 | 131 | 1.864 | 1.773 | 8.3 | 22 | A01 | 90.1 |
| P48 | Diagnostic | 716 | 1.79 | 1.861 | 7 | 24 | H01 | 13.3 |
| P48 | CR1 | 72 | 1.721 | 1.738 | 8.6 | 20.2 | G01 | 58.2 |
| P49 | Diagnostic | 1042 | 1.49 | 1.539 | 7.2 | 22.8 | B01 | 32.5 |
| P49 | CR1 | 73.2 | 1.848 | 1.419 | 8.7 | 20.2 | A01 | 45.8 |
| P4 | CR1 | 117 | 1.866 | 1.065 | 7.8 | 22.8 | G01 | 46.4 |
| P5 | Diagnostic | 784 | 1.729 | 1.794 | 8.4 | 23.6 | F02 | 36.4 |
| P50 | Diagnostic | 266 | 1.96 | 1.8 | 9.3 | 9.2 | G01 | 25.7 |
| P51 | Diagnostic | 888 | 1.644 | 1.768 | 7 | 23.6 | E01 | 52.8 |
| P5 | CR1 | 176 | 1.872 | 2.115 | 8 | 22.4 | E02 | 38 |
| P6 | Diagnostic | 665.6 | 1.821 | 1.946 | 8.2 | 26.4 | H01 | 39 |
| P6 | CR1 | 182.4 | 1.869 | 1.949 | 9.4 | 23.2 | G01 | 44 |
| P7 | Diagnostic | 114 | 1.851 | 1.931 | 8.8 | 21.2 | B02 | 58.2 |
| P8 | Diagnostic | 812 | 1.717 | 1.842 | 7 | 21.6 | C01 | 58.8 |
| P9 | Diagnostic | 996 | 1.551 | 1.588 | 5.8 | 24.4 | D02 | 20.8 |
| P9 | CR1 | 123 | 1.867 | 1.03 | 9.1 | 22.8 | C02 | 31 |

**Batch number information for sequencing runs**

**Table SIV.3 Reagent for library preparation**

Agilent SureSelect Strand Specific RNA Library Prep ILM (Agilent Technologies, Inc. Santa Clara, California)

| **Library preparation and sequencing batch number** | **Research ID** |
| --- | --- |
| Batch 1 | P18 |
| Batch 1 | P19 |
| Batch 1 | P2 |
| Batch 1 | P22 |
| Batch 1 | P3 |
| Batch 1 | P36 |
| Batch 1 | P44 |
| Batch 1 | P50 |
| Batch 2 | P10 |
| Batch 2 | P10-CR1 |
| Batch 2 | P14 |
| Batch 2 | P14- CR1 |
| Batch 2 | P25 |
| Batch 2 | P25- CR1 |
| Batch 2 | P29 |
| Batch 2 | P29- CR1 |
| Batch 2 | P31 |
| Batch 2 | P31- CR1 |
| Batch 2 | P32 |
| Batch 2 | P32- CR1 |
| Batch 2 | P37 |
| Batch 2 | P37- CR1 |
| Batch 2 | P47 |
| Batch 2 | P47- CR1 |
| Batch 2 | P48 |
| Batch 2 | P48- CR1 |
| Batch 2 | P49 |
| Batch 2 | P49- CR1 |
| Batch 2 | P4- CR1 |
| Batch 2 | P6 |
| Batch 2 | P6- CR1 |
| Batch 2 | P9 |
| Batch 2 | P9- CR1 |
| Batch 3 | P1 |
| Batch 3 | P11 |
| Batch 3 | P12 |
| Batch 3 | P13 |
| Batch 3 | P15 |
| Batch 3 | P16 |
| Batch 3 | P17 |
| Batch 3 | P20 |
| Batch 3 | P21 |
| Batch 3 | P23 |
| Batch 3 | P24 |
| Batch 3 | P26 |
| Batch 3 | P27 |
| Batch 3 | P28 |
| Batch 3 | P30 |
| Batch 3 | P33 |
| Batch 3 | P34 |
| Batch 3 | P35 |
| Batch 3 | P38 |
| Batch 3 | P39 |
| Batch 3 | P4 |
| Batch 3 | P40 |
| Batch 3 | P41 |
| Batch 3 | P42 |
| Batch 3 | P43 |
| Batch 3 | P45 |
| Batch 3 | P46 |
| Batch 3 | P5 |
| Batch 3 | P51 |
| Batch 3 | P5- CR1 |
| Batch 3 | P7 |
| Batch 3 | P8 |

**Table SIV.4 Transcriptome sequencing alignment rates and quality control before and after filtering using Fastp**

| **ID** | **Sample type** | **Before Filtering** | | | | | **After filtering with FastP** | | | | | **STAR aligner** |
| --- | --- | --- | --- | --- | --- | --- | --- | --- | --- | --- | --- | --- |
|  |  | **Total Reads (M)** | **Total Bases (G)** | **Q20 bases (G)** | **Q30 bases (G)** | **GC content** | **Total Reads (M)** | **Total Bases (G)** | **Q20 bases (G)** | **Q30 bases (G)** | **GC content** | **Rate** |
| H1 | Control | 204.937516 M | 30.740627 G | 29.710470 G (96.648872%) | 28.282158 G (92.002539%) | 51.97% | 200.582200 M | 28.491876 G | 27.807361 G (97.597510%) | 26.574192 G (93.269368%) | 51.79% | 77.75% |
| H10 | Control | 203.614822 M | 30.542223 G | 29.453675 G (96.435922%) | 28.003765 G (91.688693%) | 51.17% | 198.093874 M | 28.008473 G | 27.332547 G (97.586709%) | 26.100821 G (93.189018%) | 50.84% | 75.90% |
| H11 | Control | 204.373454 M | 30.656018 G | 29.562295 G (96.432274%) | 28.148710 G (91.821156%) | 51.72% | 198.095874 M | 28.018437 G | 27.375310 G (97.704631%) | 26.185902 G (93.459539%) | 51.38% | 75.94% |
| H12 | Control | 206.449308 M | 30.967396 G | 29.590790 G (95.554658%) | 28.062536 G (90.619618%) | 50.32% | 193.650038 M | 28.123332 G | 27.406784 G (97.452120%) | 26.128637 G (92.907328%) | 49.79% | 76.95% |
| H2 | Control | 201.217478 M | 30.182622 G | 29.011109 G (96.118586%) | 27.573164 G (91.354436%) | 51.93% | 194.256634 M | 27.328456 G | 26.667579 G (97.581725%) | 25.473215 G (93.211320%) | 51.50% | 74.78% |
| H3 | Control | 205.281658 M | 30.792249 G | 29.691863 G (96.426421%) | 28.248298 G (91.738340%) | 52.04% | 200.043270 M | 28.085864 G | 27.415274 G (97.612356%) | 26.204154 G (93.300151%) | 51.79% | 74.60% |
| H4 | Control | 201.291120 M | 30.193668 G | 29.173939 G (96.622707%) | 27.817013 G (92.128630%) | 51.64% | 196.284850 M | 27.802020 G | 27.179865 G (97.762197%) | 26.020770 G (93.593092%) | 51.38% | 75.26% |
| H5 | Control | 203.490280 M | 30.523542 G | 29.311970 G (96.030696%) | 27.815931 G (91.129433%) | 51.31% | 195.949308 M | 27.510920 G | 26.811803 G (97.458765%) | 25.581154 G (92.985456%) | 50.92% | 72.34% |
| H6 | Control | 205.398866 M | 30.809830 G | 29.730622 G (96.497195%) | 28.272751 G (91.765359%) | 51.08% | 200.116286 M | 28.327702 G | 27.640751 G (97.574987%) | 26.395680 G (93.179744%) | 50.75% | 76.74% |
| H7 | Control | 223.413880 M | 33.512082 G | 32.351991 G (96.538291%) | 30.772574 G (91.825313%) | 51.19% | 217.252512 M | 30.864005 G | 30.122312 G (97.596902%) | 28.766971 G (93.205568%) | 50.80% | 76.30% |
| H8 | Control | 206.499396 M | 30.974909 G | 29.901086 G (96.533247%) | 28.442978 G (91.825862%) | 50.15% | 201.014160 M | 28.668895 G | 27.979331 G (97.594730%) | 26.713357 G (93.178886%) | 49.78% | 78.01% |
| H9 | Control | 207.375814 M | 31.106372 G | 30.047914 G (96.597295%) | 28.679319 G (92.197571%) | 50.71% | 201.198554 M | 28.568116 G | 27.940565 G (97.803320%) | 26.783375 G (93.752683%) | 50.32% | 75.91% |
| P1 | DX | 249.373110 M | 37.405967 G | 35.615217 G (95.212663%) | 33.714648 G (90.131738%) | 50.83% | 228.084158 M | 32.345012 G | 31.483687 G (97.337070%) | 30.020257 G (92.812630%) | 49.10% | 71.20% |
| P10 | DX | 202.259964 M | 30.338995 G | 29.093796 G (95.895714%) | 27.550784 G (90.809814%) | 49.19% | 195.139656 M | 27.145768 G | 26.430231 G (97.364091%) | 25.172563 G (92.731074%) | 48.59% | 71.81% |
| P10- CR1 | CR1 | 208.673416 M | 31.301012 G | 30.172622 G (96.395035%) | 28.776218 G (91.933826%) | 51.74% | 202.312654 M | 27.867469 G | 27.255414 G (97.803696%) | 26.141167 G (93.805315%) | 51.30% | 94.11% |
| P11 | DX | 230.670052 M | 34.600508 G | 33.141853 G (95.784297%) | 31.382721 G (90.700175%) | 50.44% | 221.094238 M | 31.537893 G | 30.656469 G (97.205190%) | 29.168029 G (92.485662%) | 49.98% | 75.38% |
| P12 | DX | 205.920906 M | 30.888136 G | 30.115267 G (97.497846%) | 29.050111 G (94.049415%) | 48.43% | 201.572014 M | 28.618826 G | 28.180049 G (98.466824%) | 27.282210 G (95.329592%) | 47.98% | 77.28% |
| P13 | DX | 212.302118 M | 31.845318 G | 30.631126 G (96.187221%) | 29.105484 G (91.396432%) | 49.18% | 205.464188 M | 29.272333 G | 28.514977 G (97.412726%) | 27.209683 G (92.953585%) | 48.78% | 75.92% |
| P14 | DX | 208.325950 M | 31.248893 G | 29.933254 G (95.789809%) | 28.410505 G (90.916838%) | 49.87% | 199.499668 M | 27.907945 G | 27.217016 G (97.524256%) | 25.985206 G (93.110425%) | 49.34% | 71.72% |
| P14- CR1 | CR1 | 208.610012 M | 31.291502 G | 29.982751 G (95.817552%) | 28.405190 G (90.776052%) | 51.54% | 200.646974 M | 28.236121 G | 27.500419 G (97.394463%) | 26.191165 G (92.757657%) | 51.10% | 93.85% |
| P15 | DX | 215.924468 M | 32.388670 G | 31.022596 G (95.782245%) | 29.443502 G (90.906796%) | 49.79% | 203.941208 M | 28.985084 G | 28.242311 G (97.437397%) | 26.962163 G (93.020822%) | 48.88% | 73.68% |
| P16 | DX | 216.507110 M | 32.476067 G | 31.266722 G (96.276197%) | 29.681284 G (91.394333%) | 49.56% | 210.193486 M | 30.055105 G | 29.259461 G (97.352717%) | 27.885400 G (92.780912%) | 49.21% | 74.77% |
| P17 | DX | 251.326016 M | 37.698902 G | 36.279861 G (96.235856%) | 34.446089 G (91.371597%) | 49.75% | 243.323636 M | 34.733657 G | 33.823647 G (97.380034%) | 32.247727 G (92.842878%) | 49.37% | 93.23% |
| P18 | DX | 201.914762 M | 30.287214 G | 29.580482 G (97.666565%) | 28.622205 G (94.502599%) | 49.71% | 197.949038 M | 27.526552 G | 27.086565 G (98.401591%) | 26.319318 G (95.614293%) | 49.57% | 95.43% |
| P19 | DX | 203.548838 M | 30.532326 G | 29.818218 G (97.661143%) | 28.841797 G (94.463150%) | 51.18% | 199.444322 M | 27.791147 G | 27.348095 G (98.405781%) | 26.565794 G (95.590852%) | 51.19% | 94.78% |
| P2 | DX | 202.571450 M | 30.385717 G | 29.725044 G (97.825711%) | 28.783606 G (94.727420%) | 50.00% | 199.310860 M | 27.827664 G | 27.387781 G (98.419259%) | 26.621135 G (95.664283%) | 49.85% | 94.40% |
| P20 | DX | 266.532300 M | 39.979845 G | 38.425858 G (96.113074%) | 36.463536 G (91.204797%) | 49.83% | 257.573510 M | 36.410353 G | 35.448665 G (97.358751%) | 33.793602 G (92.813166%) | 49.30% | 74.22% |
| P21 | DX | 204.545952 M | 30.681893 G | 29.497847 G (96.140898%) | 28.011704 G (91.297184%) | 50.26% | 197.471806 M | 27.936603 G | 27.223128 G (97.446093%) | 25.975209 G (92.979127%) | 49.88% | 74.31% |
| P22 | DX | 225.031040 M | 33.754656 G | 32.955177 G (97.631500%) | 31.882488 G (94.453600%) | 50.86% | 220.698618 M | 30.316776 G | 29.838836 G (98.423515%) | 28.993591 G (95.635469%) | 50.86% | 94.54% |
| P23 | DX | 223.310758 M | 33.496614 G | 32.605514 G (97.339731%) | 31.431381 G (93.834503%) | 49.55% | 217.340612 M | 30.801792 G | 30.322428 G (98.443713%) | 29.349490 G (95.285008%) | 49.14% | 77.93% |
| P24 | DX | 221.812352 M | 33.271853 G | 32.035747 G (96.284831%) | 30.405763 G (91.385841%) | 48.61% | 215.991892 M | 30.741498 G | 29.921030 G (97.331075%) | 28.510793 G (92.743667%) | 48.14% | 76.27% |
| P25 | DX | 204.135726 M | 30.620359 G | 29.444612 G (96.160245%) | 28.016450 G (91.496152%) | 50.43% | 196.155044 M | 27.610087 G | 26.967421 G (97.672350%) | 25.798604 G (93.439052%) | 49.97% | 73.32% |
| P25- CR1 | CR1 | 201.380596 M | 30.207089 G | 29.002278 G (96.011494%) | 27.539470 G (91.168897%) | 51.19% | 192.985592 M | 27.177790 G | 26.517141 G (97.569157%) | 25.321086 G (93.168302%) | 50.72% | 93.94% |
| P26 | DX | 218.591050 M | 32.788657 G | 31.881051 G (97.231950%) | 30.699407 G (93.628129%) | 49.07% | 213.126890 M | 30.123629 G | 29.634568 G (98.376486%) | 28.648573 G (95.103324%) | 48.58% | 76.67% |
| P27 | DX | 212.181272 M | 31.827191 G | 30.596199 G (96.132264%) | 28.815086 G (90.536064%) | 47.80% | 206.444664 M | 29.188633 G | 28.395572 G (97.282982%) | 26.878665 G (92.086071%) | 47.29% | 78.31% |
| P28 | DX | 214.200028 M | 32.130004 G | 31.047402 G (96.630557%) | 29.573109 G (92.042032%) | 48.95% | 208.550712 M | 29.689894 G | 29.013795 G (97.722800%) | 27.744389 G (93.447249%) | 48.51% | 77.80% |
| P29 | DX | 212.390762 M | 31.858614 G | 30.597373 G (96.041130%) | 28.985640 G (90.982113%) | 49.17% | 205.481000 M | 28.907909 G | 28.143532 G (97.355819%) | 26.792445 G (92.682055%) | 48.64% | 73.98% |
| P29- CR1 | CR1 | 217.626170 M | 32.643926 G | 31.321139 G (95.947833%) | 29.679979 G (90.920372%) | 50.05% | 209.814550 M | 29.305132 G | 28.535951 G (97.375270%) | 27.188125 G (92.775987%) | 49.46% | 94.39% |
| P3 | DX | 206.020754 M | 30.903113 G | 30.196561 G (97.713654%) | 29.217922 G (94.546858%) | 48.31% | 202.319840 M | 28.362980 G | 27.898391 G (98.361990%) | 27.104852 G (95.564191%) | 48.11% | 95.04% |
| P30 | DX | 261.249130 M | 39.187370 G | 38.144489 G (97.338733%) | 36.693797 G (93.636795%) | 48.30% | 255.630686 M | 36.304715 G | 35.686518 G (98.297200%) | 34.457333 G (94.911457%) | 47.82% | 78.43% |
| P31 | DX | 205.950484 M | 30.892573 G | 29.579289 G (95.748871%) | 28.137440 G (91.081568%) | 50.96% | 195.439984 M | 27.394797 G | 26.761374 G (97.687798%) | 25.613616 G (93.498104%) | 50.40% | 72.73% |
| P31-CR1 | CR1 | 272.642038 M | 40.896306 G | 39.196680 G (95.844061%) | 37.233596 G (91.043912%) | 51.05% | 259.521410 M | 36.525725 G | 35.629028 G (97.545027%) | 34.041009 G (93.197353%) | 50.50% | 93.46% |
| CR1 | DX | 203.899888 M | 30.584983 G | 29.455438 G (96.306863%) | 27.973429 G (91.461319%) | 50.86% | 198.293058 M | 27.962654 G | 27.257134 G (97.476921%) | 26.001885 G (92.987900%) | 50.53% | 75.49% |
| P32-CR1 | CR1 | 200.875228 M | 30.131284 G | 28.998650 G (96.241004%) | 27.604702 G (91.614754%) | 50.07% | 193.828038 M | 27.069668 G | 26.440153 G (97.674464%) | 25.305910 G (93.484377%) | 49.60% | 94.30% |
| CR1 | DX | 207.178448 M | 31.076767 G | 29.896587 G (96.202372%) | 28.399449 G (91.384825%) | 49.72% | 200.420326 M | 28.394923 G | 27.665510 G (97.431188%) | 26.399090 G (92.971165%) | 49.30% | 75.78% |
| P34 | DX | 201.525050 M | 30.228758 G | 29.194164 G (96.577454%) | 27.795417 G (91.950247%) | 48.90% | 196.804760 M | 27.613678 G | 26.971556 G (97.674624%) | 25.791674 G (93.401808%) | 48.30% | 74.34% |
| P35 | DX | 206.796790 M | 31.019518 G | 29.777023 G (95.994471%) | 28.276073 G (91.155744%) | 50.42% | 199.006490 M | 28.096053 G | 27.372497 G (97.424706%) | 26.124406 G (92.982477%) | 50.01% | 73.93% |
| P36 | DX | 278.056358 M | 41.708454 G | 40.705151 G (97.594486%) | 39.383927 G (94.426725%) | 49.87% | 272.200750 M | 37.633964 G | 37.034462 G (98.407019%) | 35.993755 G (95.641678%) | 49.72% | 94.66% |
| P37 | DX | 202.585656 M | 30.387848 G | 29.125740 G (95.846669%) | 27.655966 G (91.009951%) | 50.96% | 194.761820 M | 26.908309 G | 26.254264 G (97.569356%) | 25.082490 G (93.214663%) | 50.37% | 71.45% |
| P37- CR1 | CR1 | 275.474456 M | 41.321168 G | 39.737026 G (96.166269%) | 37.700188 G (91.236986%) | 50.95% | 265.872260 M | 38.009421 G | 37.025341 G (97.410958%) | 35.280751 G (92.821069%) | 50.60% | 92.65% |
| P38 | DX | 222.879470 M | 33.431920 G | 32.365819 G (96.811126%) | 30.891045 G (92.399853%) | 49.60% | 218.006380 M | 30.840214 G | 30.163422 G (97.805491%) | 28.901202 G (93.712715%) | 49.11% | 93.19% |
| P39 | DX | 200.415624 M | 30.062344 G | 29.057803 G (96.658476%) | 27.726530 G (92.230102%) | 49.42% | 195.495028 M | 27.654171 G | 27.042149 G (97.786872%) | 25.908154 G (93.686243%) | 48.95% | 75.72% |
| P4 | DX | 201.628882 M | 30.244332 G | 28.669442 G (94.792774%) | 27.102095 G (89.610491%) | 52.94% | 180.793312 M | 25.460487 G | 24.807628 G (97.435796%) | 23.665395 G (92.949500%) | 50.95% | 68.79% |
| P40 | DX | 199.866414 M | 29.979962 G | 28.815459 G (96.115730%) | 27.366761 G (91.283508%) | 50.60% | 193.094310 M | 27.229471 G | 26.532120 G (97.438982%) | 25.322653 G (92.997227%) | 50.24% | 73.09% |
| P41 | DX | 261.509206 M | 39.226381 G | 38.116620 G (97.170881%) | 36.628477 G (93.377152%) | 48.84% | 255.045862 M | 36.226137 G | 35.590396 G (98.245079%) | 34.331750 G (94.770665%) | 48.38% | 77.80% |
| P42 | DX | 201.205252 M | 30.180788 G | 29.218054 G (96.810110%) | 27.862570 G (92.318897%) | 49.21% | 197.220196 M | 27.985750 G | 27.349228 G (97.725547%) | 26.176111 G (93.533710%) | 48.77% | 77.82% |
| P43 | DX | 227.866588 M | 34.179988 G | 32.904125 G (96.267221%) | 31.255742 G (91.444566%) | 49.72% | 221.195002 M | 31.273906 G | 30.465401 G (97.414761%) | 29.064608 G (92.935651%) | 49.32% | 75.99% |
| P44 | DX | 225.484502 M | 33.822675 G | 32.816072 G (97.023880%) | 31.547316 G (93.272681%) | 50.50% | 218.455034 M | 30.620997 G | 30.053204 G (98.145739%) | 29.077972 G (94.960890%) | 50.45% | 93.62% |
| P45 | DX | 204.128832 M | 30.619325 G | 29.389026 G (95.981952%) | 27.909929 G (91.151353%) | 50.25% | 196.217238 M | 27.860399 G | 27.139646 G (97.412983%) | 25.896642 G (92.951440%) | 49.79% | 74.51% |
| P46 | DX | 220.198934 M | 33.029840 G | 31.914202 G (96.622331%) | 30.390515 G (92.009272%) | 48.57% | 215.125394 M | 30.407994 G | 29.693074 G (97.648907%) | 28.390745 G (93.366057%) | 48.05% | 75.57% |
| P47 | DX | 211.292724 M | 31.693909 G | 30.495204 G (96.217870%) | 29.314938 G (92.493917%) | 49.48% | 200.866440 M | 28.219867 G | 27.724498 G (98.244608%) | 26.874861 G (95.233831%) | 49.56% | 95.02% |
| P47- CR1 | CR1 | 211.206364 M | 31.680955 G | 30.491776 G (96.246393%) | 28.942979 G (91.357662%) | 50.66% | 205.094070 M | 29.002079 G | 28.269588 G (97.474351%) | 26.956249 G (92.945921%) | 50.26% | 94.11% |
| P48 | DX | 200.867250 M | 30.130088 G | 28.863510 G (95.796302%) | 27.408386 G (90.966832%) | 54.07% | 185.795462 M | 26.645302 G | 26.011259 G (97.620435%) | 24.875569 G (93.358183%) | 52.44% | 74.01% |
| P48- CR1 | CR1 | 202.344418 M | 30.351663 G | 29.257243 G (96.394201%) | 27.861181 G (91.794579%) | 50.88% | 195.354818 M | 27.633342 G | 26.993597 G (97.684879%) | 25.827771 G (93.465969%) | 50.46% | 93.39% |
| P49 | DX | 205.239746 M | 30.785962 G | 29.587094 G (96.105795%) | 28.108674 G (91.303543%) | 51.30% | 198.105942 M | 27.989263 G | 27.296244 G (97.523983%) | 26.059946 G (93.106939%) | 50.98% | 73.03% |
| P49- CR1 | CR1 | 215.95081 M | 32.392622 G | 31.095047 G (95.994229%) | 29.461662 G (90.951767%) | 50.61% | 209.057688 M | 29.297949 G | 28.524784 G (97.361026%) | 27.164189 G (92.717032%) | 50.12% | 93.93% |
| P4- CR1 | CR1 | 204.950928 M | 30.742639 G | 29.516817 G (96.012633%) | 28.098296 G (91.398451%) | 51.15% | 195.950226 M | 27.417988 G | 26.787229 G (97.699472%) | 25.648716 G (93.547041%) | 50.68% | 91.35% |
| P5 | DX | 203.937576 M | 30.590636 G | 29.272609 G (95.691401%) | 27.775602 G (90.797724%) | 51.45% | 194.338800 M | 27.270543 G | 26.583561 G (97.480863%) | 25.374973 G (93.049020%) | 51.02% | 71.85% |
| P50 | DX | 229.632824 M | 34.444924 G | 33.550699 G (97.403899%) | 32.356620 G (93.937267%) | 50.68% | 224.573894 M | 31.102466 G | 30.572139 G (98.294902%) | 29.640837 G (95.300601%) | 50.66% | 94.13% |
| P51 | DX | 246.574166 M | 36.986125 G | 35.615580 G (96.294434%) | 33.869427 G (91.573332%) | 49.86% | 238.605826 M | 33.926449 G | 33.075850 G (97.492813%) | 31.590145 G (93.113619%) | 49.45% | 76.28% |
| P5- CR1 | CR1 | 200.817682 M | 30.122652 G | 28.929643 G (96.039493%) | 27.528280 G (91.387305%) | 52.17% | 192.121096 M | 27.024436 G | 26.397094 G (97.678610%) | 25.261382 G (93.476074%) | 51.80% | 92.42% |
| P6 | DX | 203.112924 M | 30.466939 G | 29.186929 G (95.798692%) | 27.669248 G (90.817289%) | 48.94% | 192.189950 M | 26.800957 G | 26.132846 G (97.507138%) | 24.941123 G (93.060568%) | 47.64% | 70.57% |
| P6- CR1 | CR1 | 226.914764 M | 34.037215 G | 32.744729 G (96.202730%) | 31.084695 G (91.325615%) | 50.25% | 219.914366 M | 30.754559 G | 29.985357 G (97.498902%) | 28.612051 G (93.033529%) | 49.76% | 94.50% |
| P7 | DX | 206.569038 M | 30.985356 G | 29.620512 G (95.595197%) | 27.899664 G (90.041453%) | 48.31% | 200.612776 M | 28.353421 G | 27.444620 G (96.794742%) | 25.963168 G (91.569792%) | 47.71% | 74.06% |
| P8 | DX | 240.074064 M | 36.011110 G | 34.640285 G (96.193329%) | 32.889685 G (91.332050%) | 49.11% | 231.835352 M | 32.925121 G | 32.069789 G (97.402191%) | 30.582207 G (92.884114%) | 48.60% | 75.14% |
| P9 | DX | 203.494618 M | 30.524193 G | 29.480363 G (96.580320%) | 28.074401 G (91.974263%) | 54.20% | 198.253788 M | 28.509664 G | 27.812326 G (97.554030%) | 26.578573 G (93.226540%) | 54.11% | 78.91% |
| P9- CR1 | CR1 | 225.041306 M | 33.756196 G | 32.460215 G (96.160760%) | 30.837721 G (91.354253%) | 52.08% | 216.933088 M | 30.662658 G | 29.919220 G (97.575430%) | 28.567835 G (93.168162%) | 51.80% | 93.92% |

Fastp was used to perform quality control, adapter trimming, quality filtering, per-read quality pruning, and per-read polyG tail trimming.

STAR aligner was utilised to map RNA-seq data for all the pipelines in this study.

Control refers to healthy Control (H1-H12), DX refers to diagnostic samples (DX1-DX51), and CR1 refers to samples obtained from patients who attained CR1.

**MA-PLOTs for the DEG profiles**


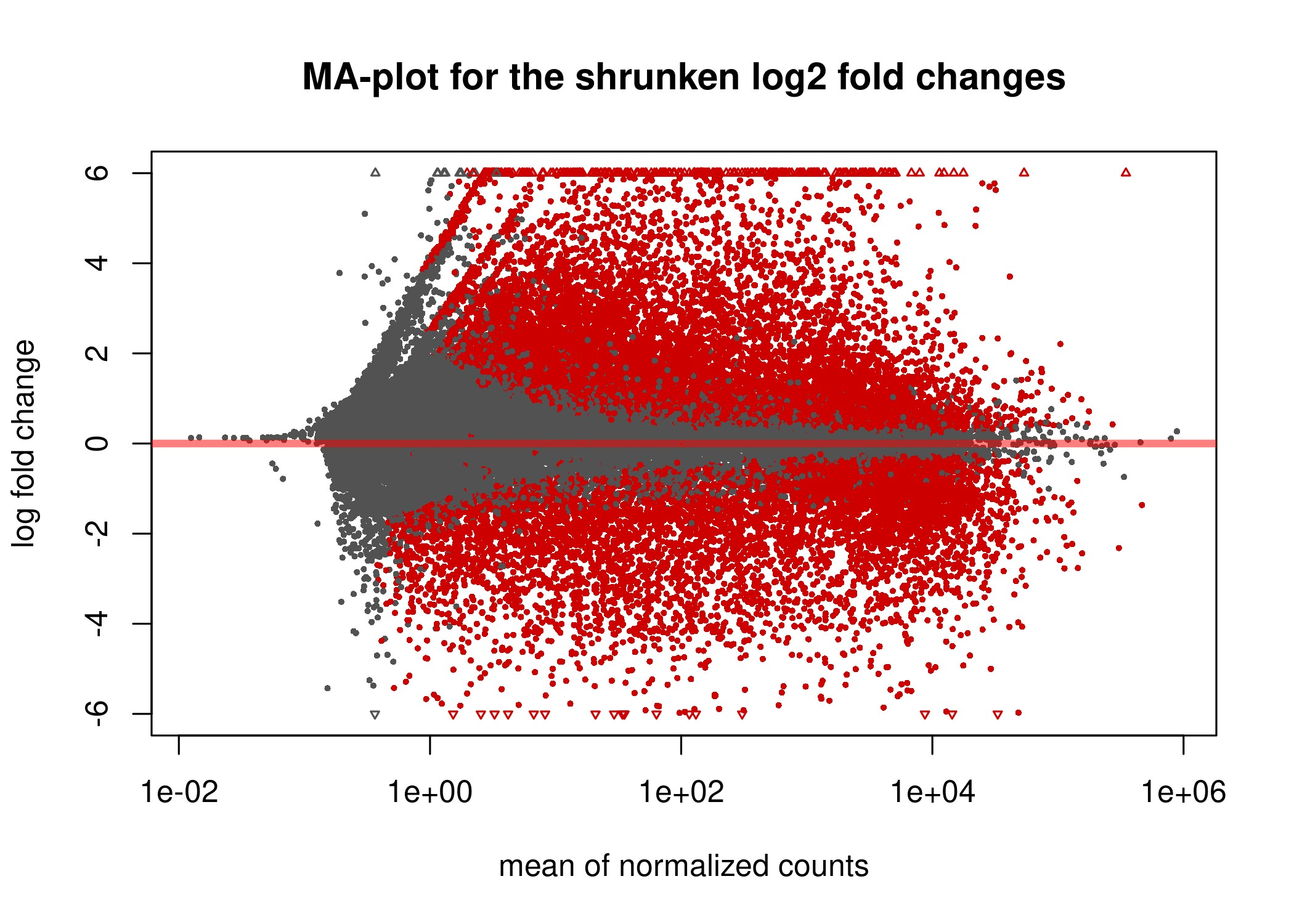


Figure SIV.1 MA-plot illustrating the overview between the AML-NK patients (n=51) and healthy control groups (n=12). On the x-axis, mean normalised counts, and on the y-axis, log2 fold changes are displayed. Red dots indicate the significantly differentially expressed points.


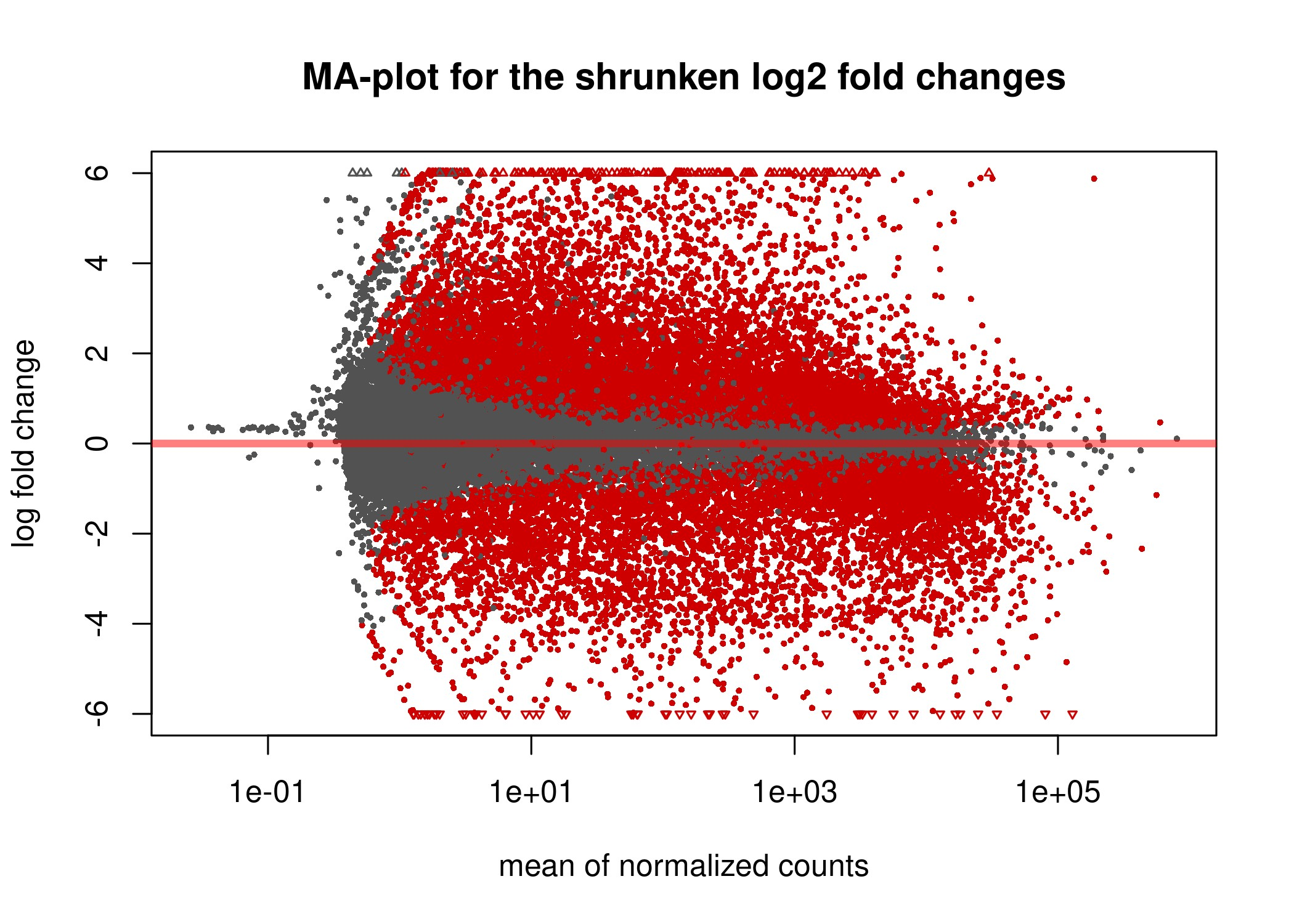


Figure SIV.2 MA-plot illustrating the overview between the AML-NK DX and CR1 samples (n=12). On the x-axis, mean normalised counts and on the y-axis, log2 fold changes are displayed. Red dots indicate the significantly differentially expressed points.


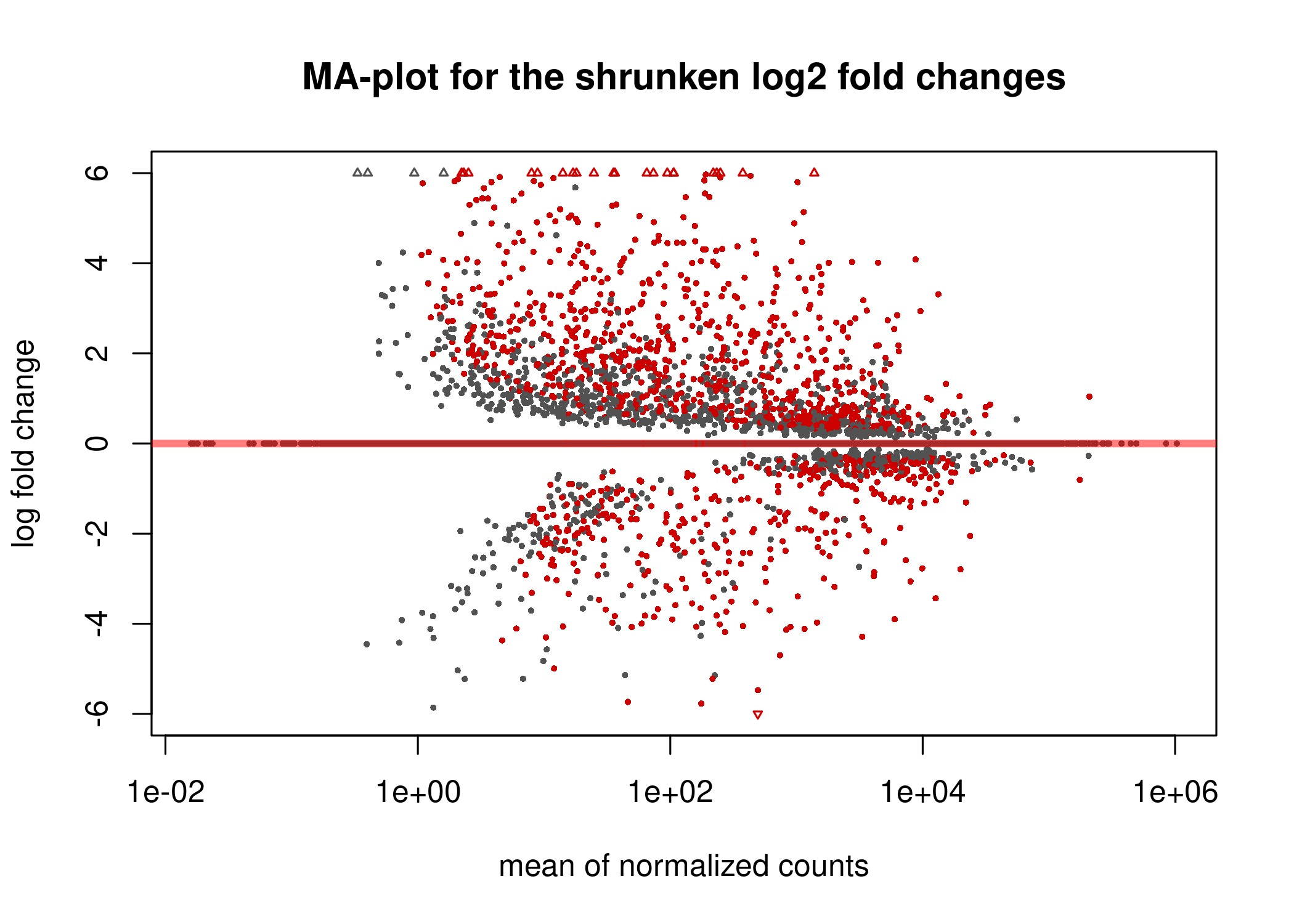


Figure SIV.3 MA-plot illustrating the overview between the AML-NK patients with genotype *FLT3*-ITD/*NPM1*^mut^  and the *FLT3*^wt^/*NPM1*^wt^. The AML-NK patients with genotype *FLT3*-ITD/*NPM1*^mut^ (n= 9) and the *FLT3*^wt^/*NPM1*^wt^ (n = 22). On the x-axis, mean normalised counts, and on the y-axis, log2 fold changes are displayed. Red dots indicate the significantly differentially expressed points.


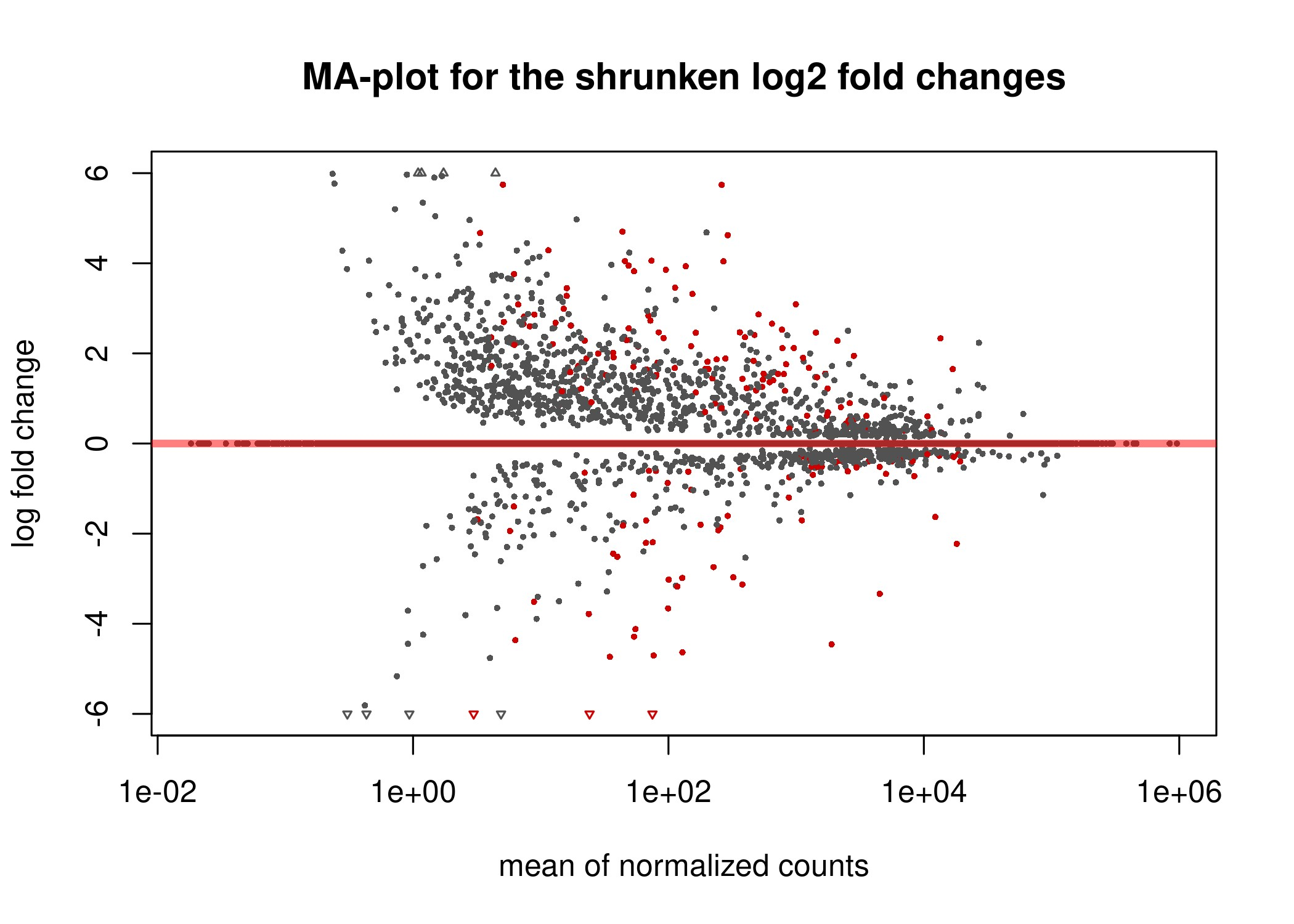


Figure SIV.4 MA-plot illustrating the overview between the AML-NK patients with OS <5 years and OS >5 years. On the x-axis, mean normalised counts, and on the y-axis, log2 fold changes are displayed. Red dots indicate the significantly differentially expressed points.
