# Supplementary material for: Gene expression profiling and pathway analysis in acute myeloid leukaemia-normal karyotype patients
Source: PLoS One. 2025 Sep 5;20(9):e0328911. doi: 10.1371/journal.pone.0328911 (PMC12412999; doi:10.1371/journal.pone.0328911)
Supplement: S7 File — (DOCX) [file pone.0328911.s007.docx]

### S VII The principal component analysis (PCA), volcano plot and a hierarchical clustering heatmap of AML-NK vs healthy controls

**AML-NK patients vs healthy controls**


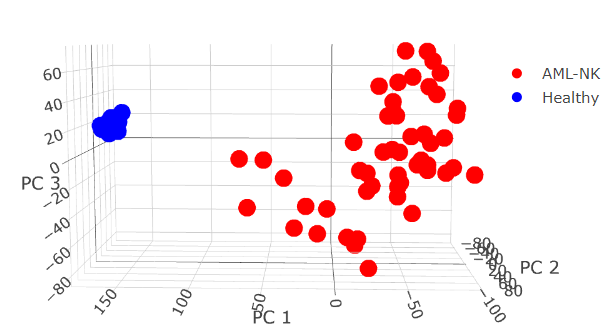


Figure SVII.1 PCA plot of AML-NK patients vs healthy controls.

AML-NK patients (n=51), red dots and healthy controls (n=12, blue dots). PC1 refers to principal component 1, PC2 refers to principal component 2, and PC3 refers to principal component 3.


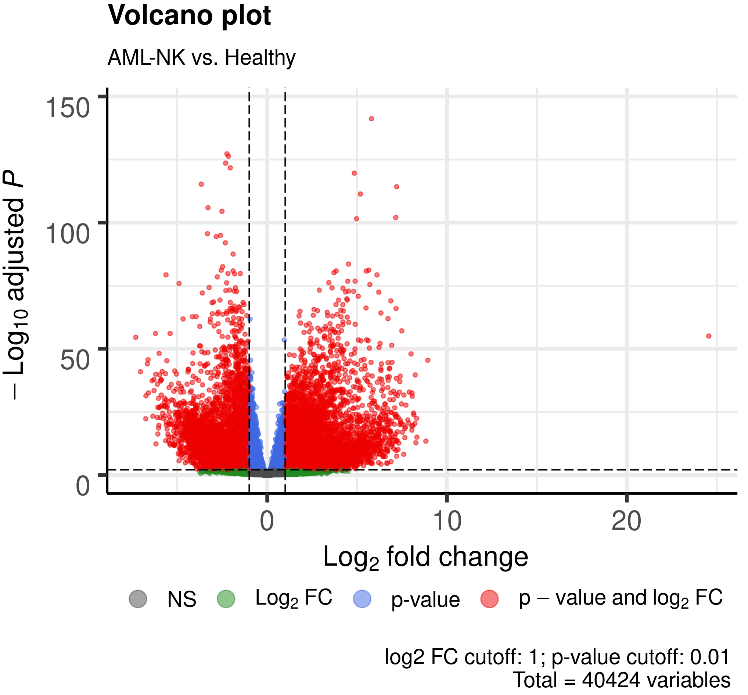


Figure SVII.2 Volcano plots for DEG profiling between AML-NK patients vs healthy groups.

The volcano plot shows a log_2_ fold change between AML-NK patients vs healthy groups on the x-axis and –log10 (p-values) on the y-axis, depicting the magnitude of fold changes between the AML-NK patients (n=51) and healthy control groups (n=12). Grey indicates a statistically insignificant change in the differential gene expression (NS), green dots indicate that only the log_2_ fold cutoff was fulfilled, blue indicates only –log10 (p values) cutoff was fulfilled, and red indicates significantly expressed genes that met the cutoff for the log_2_ fold change and –log10 (p values).


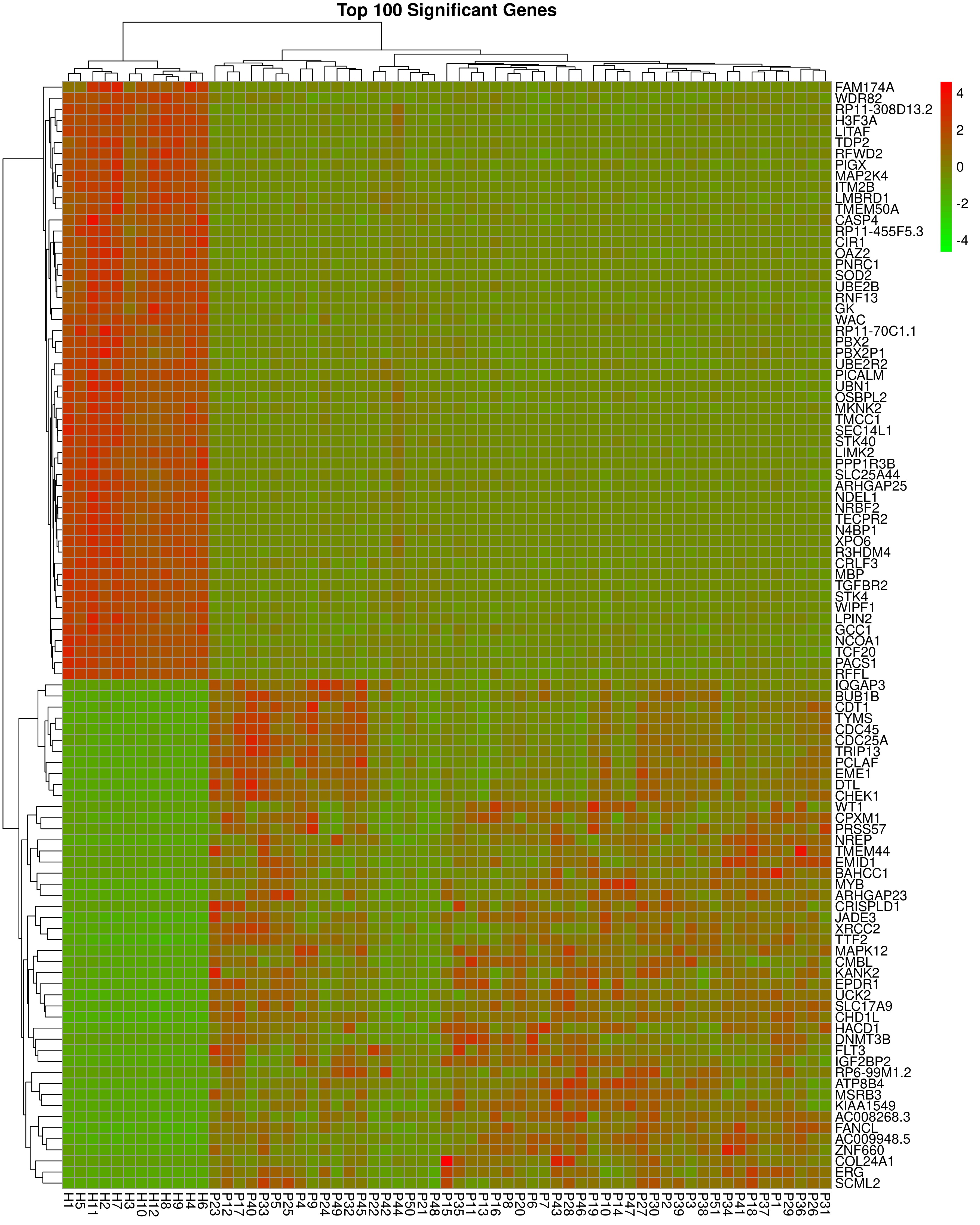


Figure SVII.3 Hierarchical clustering heatmap of AML-NK patients and healthy controls.

The heatmap depicts the correlations between the condition (AML-NK patients, n=51) and the reference (healthy controls, n=12) by the colour coding gradient between green indicating downregulation and red indicating upregulation. Variance-stabilising transformation (vst) was used on the normalised counts. P1-51 refers to AML-NK patients, and H1-12 refers to the healthy controls.
