# Supplementary material for: Gene expression profiling and pathway analysis in acute myeloid leukaemia-normal karyotype patients
Source: PLoS One. 2025 Sep 5;20(9):e0328911. doi: 10.1371/journal.pone.0328911 (PMC12412999; doi:10.1371/journal.pone.0328911)
Supplement: S9 File — (DOCX) [file pone.0328911.s009.docx]

### S IX Most significantly enriched pathways in AML-NK (DX-CR1)

**Table IX.1 Most enriched pathways (KEGG) in AML-NK versus Healthy controls**

The significant pathways are listed based on the p-value and FDR.

geneSet refers to gene sets used in the GSEA enrichment analysis; hsa refers to homo sapiens pathway; ES refers to enrichment scores; NES refers to normalised enrichment scores; size refers to the number of genes enriched in the pathway; userId refers to gene symbols.
