# Supplementary material for: Gene expression profiling and pathway analysis in acute myeloid leukaemia-normal karyotype patients
Source: PLoS One. 2025 Sep 5;20(9):e0328911. doi: 10.1371/journal.pone.0328911 (PMC12412999; doi:10.1371/journal.pone.0328911)
Supplement: S10 File — (DOCX) [file pone.0328911.s010.docx]

### S X The principal component analysis (PCA), volcano plot and a hierarchical clustering heatmap of AML-NK (DX-CR1)


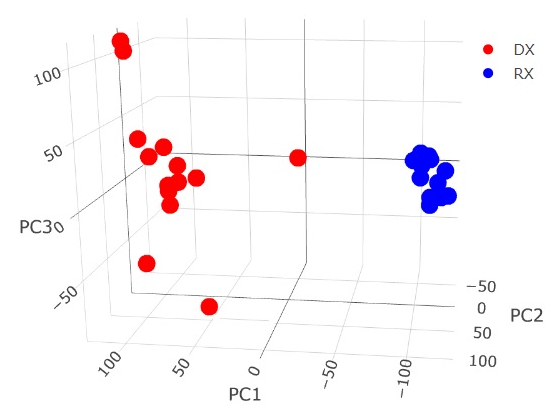


Figure SX.1 PCA plot of AML-NK patient (DX versus CR1) without outliers.

The AML-NK patient details: DX (n=12, red dots) and their CR1 samples (n=12, blue dots). Two outliers were identified in patients P4 (RX13) and P5 (RX14) and were removed in subsequent analysis. PC1 refers to principal component 1, PC2 refers to principal component 2, and vst refers to variance stabilising transformation.


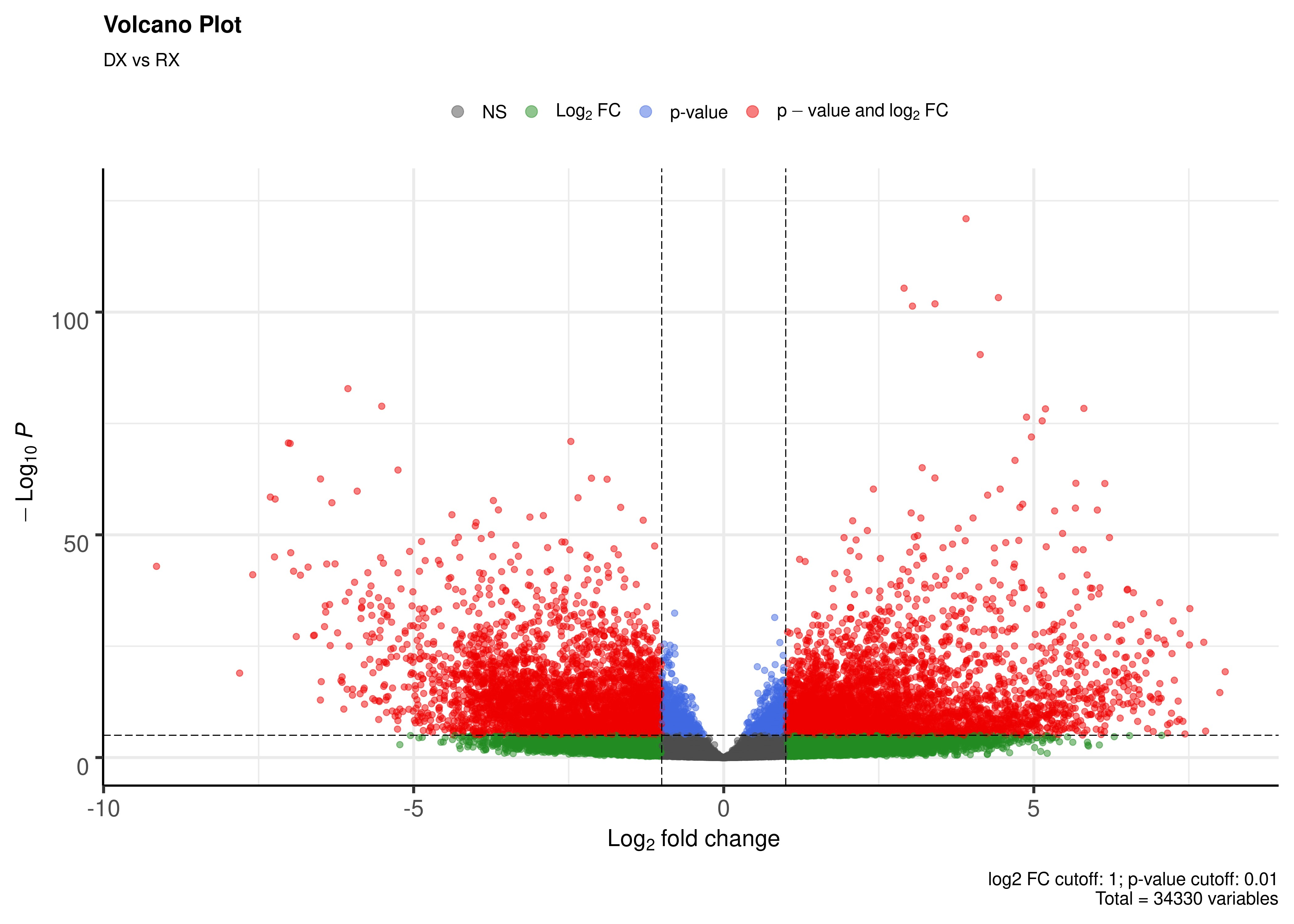


Figure SX.2 Volcano plots for DEG profiling between AML-NK DX versus CR1.

The volcano plot shows a log_2_ fold change between the AML-NK DX and CR1. Samples on the x-axis and –log10 (p-values) on the y-axis depict the magnitude of fold changes between the AML-NK DX and CR1 samples (n=12). Grey indicates a statistically insignificant change in the differential gene expression (NS), green dots indicate that only the log_2_ fold cutoff was fulfilled, blue indicates only –log10 (p values) cutoff was fulfilled, and red indicates significantly expressed genes that met the cutoff for the log_2_ fold change and –log10 (p values).


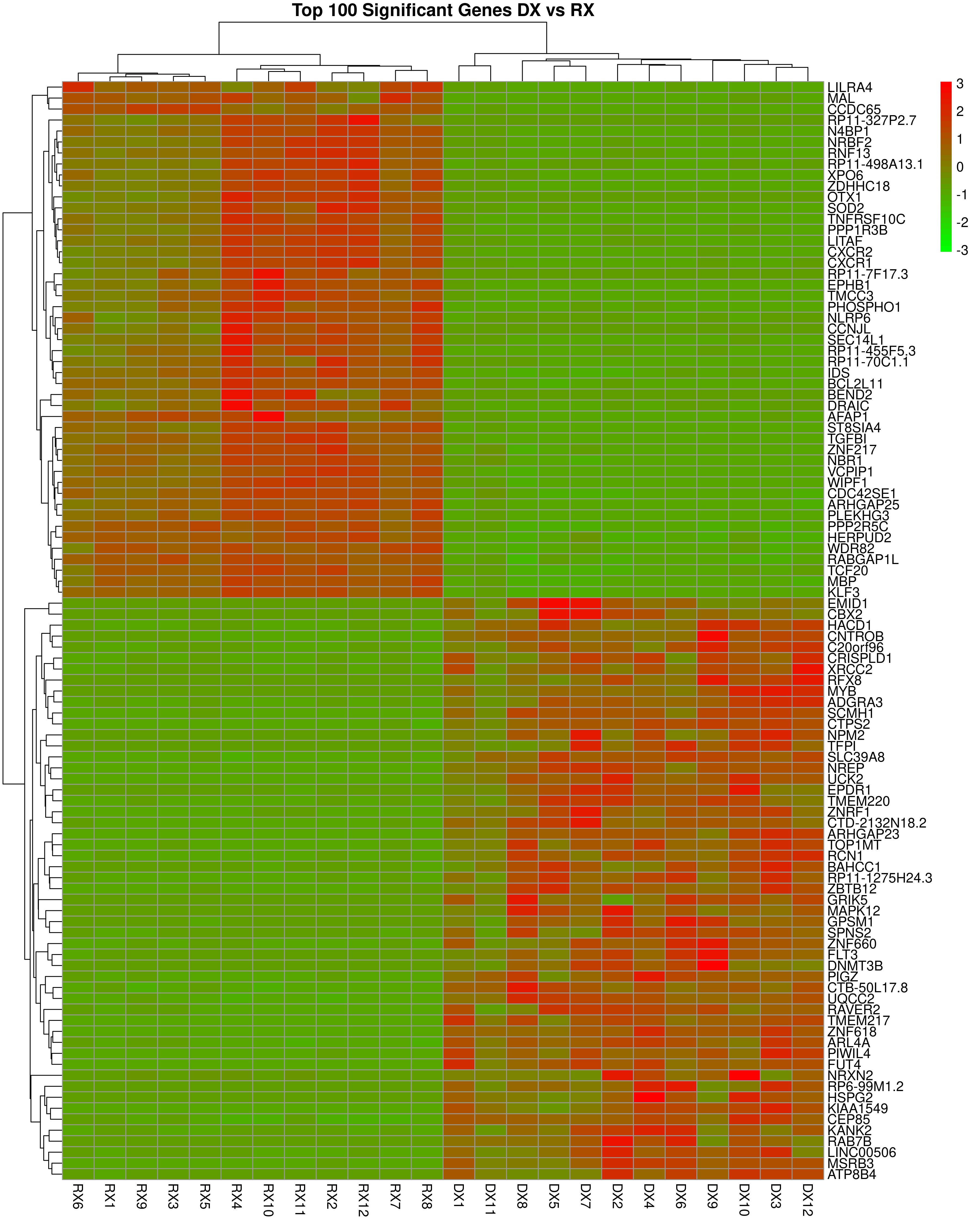


SX.3 Hierarchical clustering heatmap of AML-NK DX and CR1 samples.

The heat map depicts the correlations between the condition (DX) and their CR1 samples by the colour-coded gradient between green, indicating downregulation, and red, indicating upregulation. Variance-stabilising transformation (vst) was used on the normalised counts.
