# Supplementary material for: Gene expression profiling and pathway analysis in acute myeloid leukaemia-normal karyotype patients
Source: PLoS One. 2025 Sep 5;20(9):e0328911. doi: 10.1371/journal.pone.0328911 (PMC12412999; doi:10.1371/journal.pone.0328911)
Supplement: S11 File — (DOCX) [file pone.0328911.s011.docx]

### S XI Top 100 significant DEGS between AML-NK (DX-CR1)

**Table SXI.1 Top 100 DEGs between AML-NK DX vs CR1**

**Table SXI.1 Top 100 DEGs between AML-NK DX vs CR1 (continued)**

**Table SXI.2 Most enriched pathways (KEGG) in AML-NK DX versus CR1**

The significant pathways are listed based on the p-value and FDR.
