# Supplementary material for: Gene expression profiling and pathway analysis in acute myeloid leukaemia-normal karyotype patients
Source: PLoS One. 2025 Sep 5;20(9):e0328911. doi: 10.1371/journal.pone.0328911 (PMC12412999; doi:10.1371/journal.pone.0328911)
Supplement: S12 File — (DOCX) [file pone.0328911.s012.docx]

### S XII The RT-qPCR findings of AML-NK vs healthy controls DEGs

**The RT-qPCR findings of the *TPD2* gene**

Out of the six DEGs, the RT-qPCR findings of the *TPD2* gene, downregulated in the patient group compared to the healthy control group, were selected to describe the results as the most significantly deregulated gene based on the padj value. The annealing temperatures around the calculated Tm of the primer were tested, and the efficiency of the primers was verified by a standard curve of serial dilutions of template cDNA, as shown in Figure 6.33-Figure 6.36. Table 6.3 outlines the CT values for the *TDP2*, *B2M,* and *GAPDH* genes. The fold-change and p-values of *TDP2, B2M,* and *GAPDH* genes are summarised in Table 6.4. The fold change (2^-∆∆Ct^) between the control and patient samples is represented in a bar chart that displays that *TDP2* was significantly downregulated in the AML-NK patients compared to the healthy control group (unpaired two-tailed t-test) p-value =0.00017) (Figure 6.37).

**
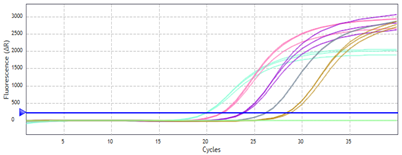
**

Figure SXII.1 *TDP2* primer performance amplification curve.

**
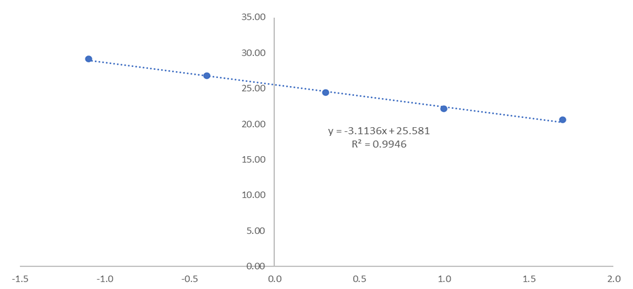
**

Figure SXII.2 *TDP2* primer efficiency standard curve. A standard curve was generated using five points with R2> 0.95 and primer efficiency >100%.


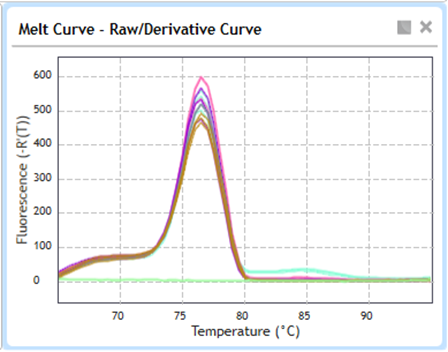


Figure SXII.3 Melt curve quality of *TDP2* gene. Smooth melt curves with only one peak in all samples were detected.

**
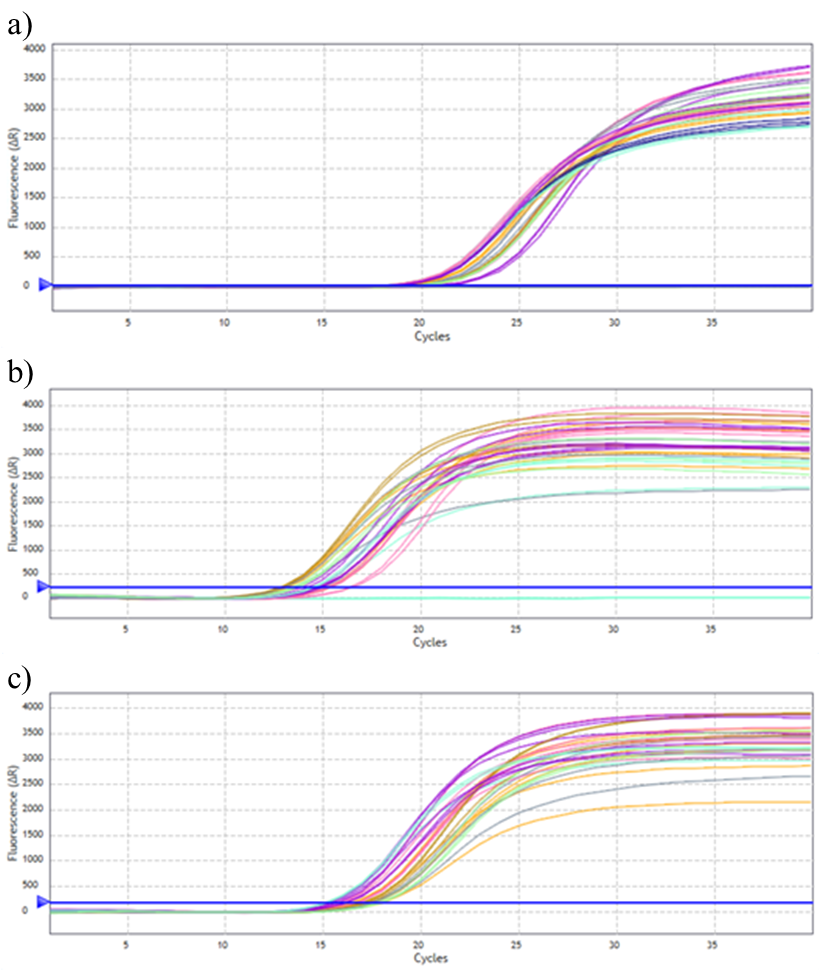
**

Figure SXII.4 Amplification curves of a) *TDP2*, b) *B2M*, and c) *GAPDH* (housekeeping) genes.

Table SXII.1 Table CT value summary for *TDP2*, *B2M*, and *GAPDH* genes.

| **ID** | ***TDP2* (GOI)** | | ***B2M* (HG)** | | ***GAPDH* (HG)** | |
| --- | --- | --- | --- | --- | --- | --- |
|  | **CT** | **Average CT** | **CT** | **Average CT** | **CT** | **Average CT** |
| P45 | 21.00 | 20.95 | 15.34 | 15.26 | 16.75 | 16.88 |
|  | 20.94 |  | 15.51 |  | 16.73 |  |
|  | 20.90 |  | 14.94 |  | 17.17 |  |
| P9 | 21.79 | 21.86 | 16.50 | 16.22 | 16.98 | 16.89 |
|  | 21.74 |  | 15.50 |  | 16.80 |  |
|  | 22.06 |  | 16.66 |  | 16.88 |  |
| P32 | 20.42 | 20.49 | 14.97 | 15.10 | 15.37 | 15.40 |
|  | 20.42 |  | 15.47 |  | 15.45 |  |
|  | 20.62 |  | 14.86 |  | 15.37 |  |
| P30 | 20.87 | 20.87 | 15.65 | 15.73 | 16.35 | 16.17 |
|  | 20.87 |  | 15.79 |  | 15.99 |  |
|  | 20.86 |  | 15.76 |  | 16.16 |  |
| P38 | 20.92 | 20.92 | 14.32 | 14.79 | 16.35 | 16.23 |
|  | 20.93 |  | 14.64 |  | 15.98 |  |
|  | 20.91 |  | 15.40 |  | 16.35 |  |
| P7 | 19.91 | 19.94 | 14.91 | 14.86 | 15.53 | 15.57 |
|  | 19.96 |  | 14.74 |  | 15.75 |  |
|  | 19.94 |  | 14.92 |  | 15.44 |  |
| H2 | 19.45 | 19.49 | 13.96 | 13.57 | 17.59 | 17.80 |
|  | 19.61 |  | 13.46 |  | 17.72 |  |
|  | 19.42 |  | 13.28 |  | 18.09 |  |
| H6 | 19.25 | 19.34 | 13.36 | 13.28 | 17.44 | 17.31 |
|  | 19.41 |  | 13.27 |  | 17.23 |  |
|  | 19.35 |  | 13.22 |  | 17.27 |  |
| H11 | 19.30 | 19.24 | 14.12 | 13.98 | 18.08 | 18.02 |
|  | 19.24 |  | 14.01 |  | 17.76 |  |
|  | 19.19 |  | 13.82 |  | 18.21 |  |
| H12 | 19.61 | 19.63 | 13.57 | 13.45 | 18.24 | 17.92 |
|  | 19.62 |  | 13.42 |  | 17.73 |  |
|  | 19.65 |  | 13.35 |  | 17.80 |  |
| NTC | No Cq | No Cq | No Cq | No Cq  No Cq  No Cq | No Cq | No Cq |
|  | No Cq |  | No Cq |  | No Cq |  |
|  | No Cq |  | No Cq |  | No Cq |  |

GOI: gene of interest (*TDP2*)

HG: housekeeping genes (*B2M* and *GAPDH*)

∆CT: delta CT (difference between CT value between GOI and HG)

Cq: quantification cycle

Control IDs begin with H, and AML-NK patient ID begins with and is followed by a numeric code.

Table SXII.2 Fold change and p-values of *TDP2* and *B2M* and *GAPDH* genes

| **Type** | **Average GOI CT** | **Average HG CT** | **∆CT** | **Average ∆CT** | **∆∆CT** | **Fold Change (2^-∆∆Ct^)** | **SD** |
| --- | --- | --- | --- | --- | --- | --- | --- |
| H2 | 19.49 | 15.68 | 3.81 | 3.76 | 0.00 | 1.0 | 0.356 |
| H6 | 19.34 | 15.30 | 4.04 |  |  |  |  |
| H11 | 19.24 | 16.00 | 3.24 |  |  |  |  |
| H12 | 19.63 | 15.69 | 3.94 |  |  |  |  |
| P45 | 20.95 | 16.07 | 4.87 | 5.08 | 1.32 | 0.4 | 0.278 |
| P9 | 21.86 | 16.55 | 5.31 |  |  |  |  |
| P32 | 20.49 | 15.25 | 5.24 |  |  |  |  |
| P30 | 20.87 | 15.95 | 4.92 |  |  |  |  |
| P38 | 20.92 | 15.51 | 5.41 |  |  |  |  |
| P7 | 19.94 | 15.22 | 4.72 |  |  |  |  |

GOI: gene of interest (*TDP2*)

HG: housekeeping genes (*B2M* and *GAPDH*)

CT: cycle threshold value

∆CT: delta CT (difference between CT value between GOI and HG)

∆∆CT: delta delta CT

(2^-∆∆Ct^): 2(-Delta Delta C(T))

Control IDs begin with H, and AML-NK patient ID begins with and is followed by a numeric code.

**
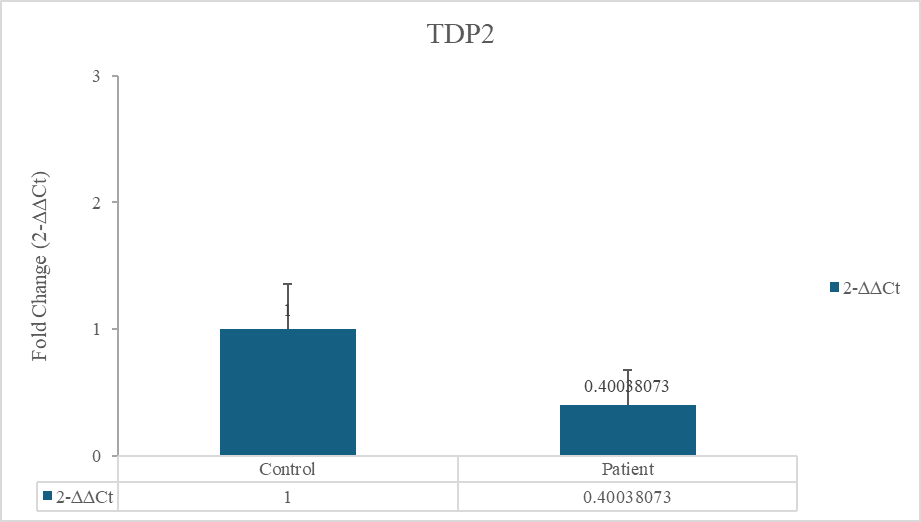
**

Figure SXII.3 Bar chart showing fold change (2^-∆∆Ct^) between the control and AML-NK patient samples.

*TDP2* was significantly downregulated in the patient group compared to the healthy control group (unpaired two-tailed t-test, p-value =0.00017).
